# Effect of medications on prevention of secondary osteoporotic vertebral compression fracture, non-vertebral fracture, and discontinuation due to adverse events: a meta-analysis of randomized controlled trials

**DOI:** 10.1186/s12891-019-2769-8

**Published:** 2019-08-31

**Authors:** Yuan-Zhe Jin, Jae Hyup Lee, Bin Xu, Minjoon Cho

**Affiliations:** 1 Department of Orthopedic Surgery, College of Medicine, Seoul National University, Seoul, 110-799 South Korea; 2 The First Hospital of Jilin University, Changchun City, 130021 China; 3 Department of Orthopedic Surgery, SMG-SNU Boramae Medical Center, Seoul, 156-707 South Korea; 4 Institute of Medical and Biological Engineering, Seoul National University Medical Research Center, Seoul, 110-799 South Korea; 5 Department of Orthopedic Surgery, Tianjin Hospital, Tianjin University, Tianjin, China

**Keywords:** Secondary prevention, Conservative treatment, Osteoporosis, Meta-analysis, Osteoporotic fracture, Senior

## Abstract

**Background:**

Bone loss with aging and menopause increases the risk of fragile vertebral fracture, osteoporotic vertebral compression fracture (OVCF). The fracture causes severe pain, impedes respiratory function, lower the quality of life, and increases the risk of new fractures and deaths. Various medications have been prescribed to prevent a secondary fracture, but few study summarized their effects. Therefore, we investigated their effects on preventing subsequent OVCF via meta-analyses of randomized controlled trials.

**Methods:**

Electronic databases, including MEDLINE, EMBASE, CENTRAL, and Web of Science were searched for published randomized controlled trials from June 2015 to June 2019. The trials that recruited participants with at least one OVCF were included. We assessed the risk of bias of every study, estimated relative risk ratio of secondary OVCF, non-vertebral fracture, gastrointestinal complaints and discontinuation due to adverse events. Finally, we evaluated the quality of evidence.

**Results:**

Forty-one articles were included. Moderate to high quality evidence proved the effectiveness of zoledronate (Relative Risk, RR: 0.34; 95% CI, 0.17–0.69, *p* = 0.003), alendronate (RR: 0.54; 95% CI: 0.43–0.68; *p* < 0.0001), risedronate (RR: 0.61; 95% CI: 0.51–0.73; *p* < 0.0001), etidronate (RR, 0.50; 95% CI, 0.29–0.87, *p* < 0.01), ibandronate (RR: 0.52; 95% CI: 0.38–0.71; *p* < 0.0001), parathyroid hormone (RR: 0.31; 95% CI: 0.23–0.41; *p* < 0.0001), denosumab (RR, 0.41; 95% CI, 0.29–0.57; *p* < 0.0001) and selective estrogen receptor modulators (Raloxifene, RR: 0.58; 95% CI: 0.44–0.76; *p* < 0.0001; Bazedoxifene, RR: 0.66; 95% CI: 0.53–0.82; *p* = 0.0002) in preventing secondary fractures. Moderate quality evidence proved romosozumab had better effect than alendronate (Romosozumab vs. alendronate, RR: 0.64; 95% CI: 0.49–0.84; *p* = 0.001) and high quality evidence proved that teriparatide had better effect than risedronate (risedronate vs. teriparatide, RR: 1.98; 95% CI: 1.44–2.70; *p* < 0.0001).

**Conclusion:**

Zoledronate, alendronate, risedronate, etidronate, ibandronate, parathyroid hormone, denosumab and selective estrogen receptor modulators had significant secondary prevention effects on OVCF. Moderate quality evidence proved romosozumab had better effect than alendronate. High quality evidence proved PTH had better effect than risedronate, but with higher risk of adverse events.

**Electronic supplementary material:**

The online version of this article (10.1186/s12891-019-2769-8) contains supplementary material, which is available to authorized users.

## Background

Osteoporotic vertebral compression fracture (OVCF) is one of the most common fragile fractures, with a prevalence of 30 to 50% in people over 50 years of age [1]. It causes severe pain and disability, raises the risk of secondary fracture more than 4-fold [2, 3], and increases the risk of mortality [4]. Therefore, secondary prevention of OVCF was critical and should be emphasized to improve patients’ quality of life. However, though the primary prevention efficacy of medications have been well summarized [5–10], only one systematic review targeted on their secondary prevention effects [11]. Therefore, to investigate the efficacy of current medication therapies on preventing secondary OVCF, we conducted this study through systematically literature review and meta-analyses of randomized controlled trials (RCTs).

## Methods

### Search for studies

Four major electronic databases (MEDLINE, EMBASE, CENTRAL, and Web of Science) were searched with a developed search strategy that consisted of keywords “controlled trials”, “osteoporotic fracture”, “bisphosphonate”, “parathyroid hormone”, “denosumab” “calcitonin”, “Raloxifene”, “Bazedoxifene” “hormone replacement”, “romosozumab”, “abaloparatide”, etc., and others (Additional file 1). The search spanned the period from June 2015 to June 2019, with weekly alerts of updated published trials. Reference lists from other reviews and studies were also checked for relevant articles. The references were managed with Endnote X7 (Clarivate Analytics).

### Selection of studies

Three authors (YZJ, BX, and MJC) independently screened the titles and abstracts of studies and evaluated their relevance to our study. A study was included if it involved patients with osteoporosis. A subsequent full-text assessment was done by three authors (YZJ, BX, and JHL) independently. Randomized controlled trials (RCTs) published in English that investigated the efficacy of currently approved medications for patients with OVCF were included. The studies that included osteoporosis patients without distinguishing their fracture history were included if the data of the participants with prevalent fractures was adequately presented. Studies that recruited patients with traumatic vertebral fracture, secondary osteoporosis, or did not report results in dichotomous data (i.e., patient-years, etc.), were excluded. The included medications were the approved ones, including zoledronate, alendronate, risedronate, etidronate, ibandronate, minodronate, pamidronate, calcitonin, hormone replacement therapy, parathyroid hormone, denosumab, romosozumab, raloxifene, and bazedoxifene [12–14]. Post hoc analyzed RCTs were also included, with taking care of duplicated data input. Disagreements between reviewers were resolved by discussion or, if unresolved, by consultation with consultation with librarians and a statistic professor from SMG-SNU Boramae Medical Center.

### Data extraction and risk of bias

Basic characteristics of each study were independently extracted by YZJ, BX, and JHL with a designed table that contains the number of participants, interventions, comparisons, and outcomes. The primary outcome of this study was the vertebral fracture ratio in the final visit, and the secondary outcomes were gastrointestinal (GI) complaints of bisphosphonates, discontinuation due to adverse events (AEs), and non-vertebral fracture ratio.

The risk of bias was measured independently by YZJ, BX, and JHL with the tool recommended in updated guidelines of Cochrane Back and Neck Group [15]. The detection bias was rated for main result (vertebral fracture). The loss ratio was acceptable for a middle- or long-term trial (observational period > 1 year), if that was not exceeded 30%. The risk of other sources of bias was rated as low risk if the article stated both conflict of interest and sponsor of the trial and no other serious risk of bias was reported.

### Data analysis and quality of evidence

Relative risk (RR) and its 95% confidence intervals (CIs) were used to estimate the effect of interventions, with *p*-values < 0.05 considered significant. The overall effect size was calculated with a random effects model [15]. Heterogeneity between studies was identified and measured with *p*-value and I^2^ value from Chi-squared test, *p*-value < 0.1 was identified as significant, and I^2^ < 40% was considered as not important, I^2^ between 40 and 74% indicated moderate to substantial, I^2^ > 75% was identified as a considerable magnitude [15]. In studies with more than two arms, intervention groups were input into each subgroup and the data in the control groups were separated equally and then were compared to their counterparts. Sensitivity analyses were used to explore the interference from a study by excluding it from syntheses and the impact from loss to follow-up population by compositing the missing events according to event ratio in control groups [16]. The data was analyzed by two authors (YZJ and JHL) with RevMan 5.3.3 (Cochrane).

We evaluated five factors of the results to determine the quality of evidence, including study limitation, imprecision, indirectness, inconsistent and publication bias, followed the GRADE approach. The criteria for downregulating the level referred to the handbook of GRADE and guidelines from Cochrane Back and Neck Group [15, 16]. In the case that an outcome included one trial with no unclear or high risk of bias, the study limitation item was rated as not serious if its result remained same direction and signficancy with the pooled result.

## Results

### Characteristics of included studies and risk of bias

**Fig. 1:**
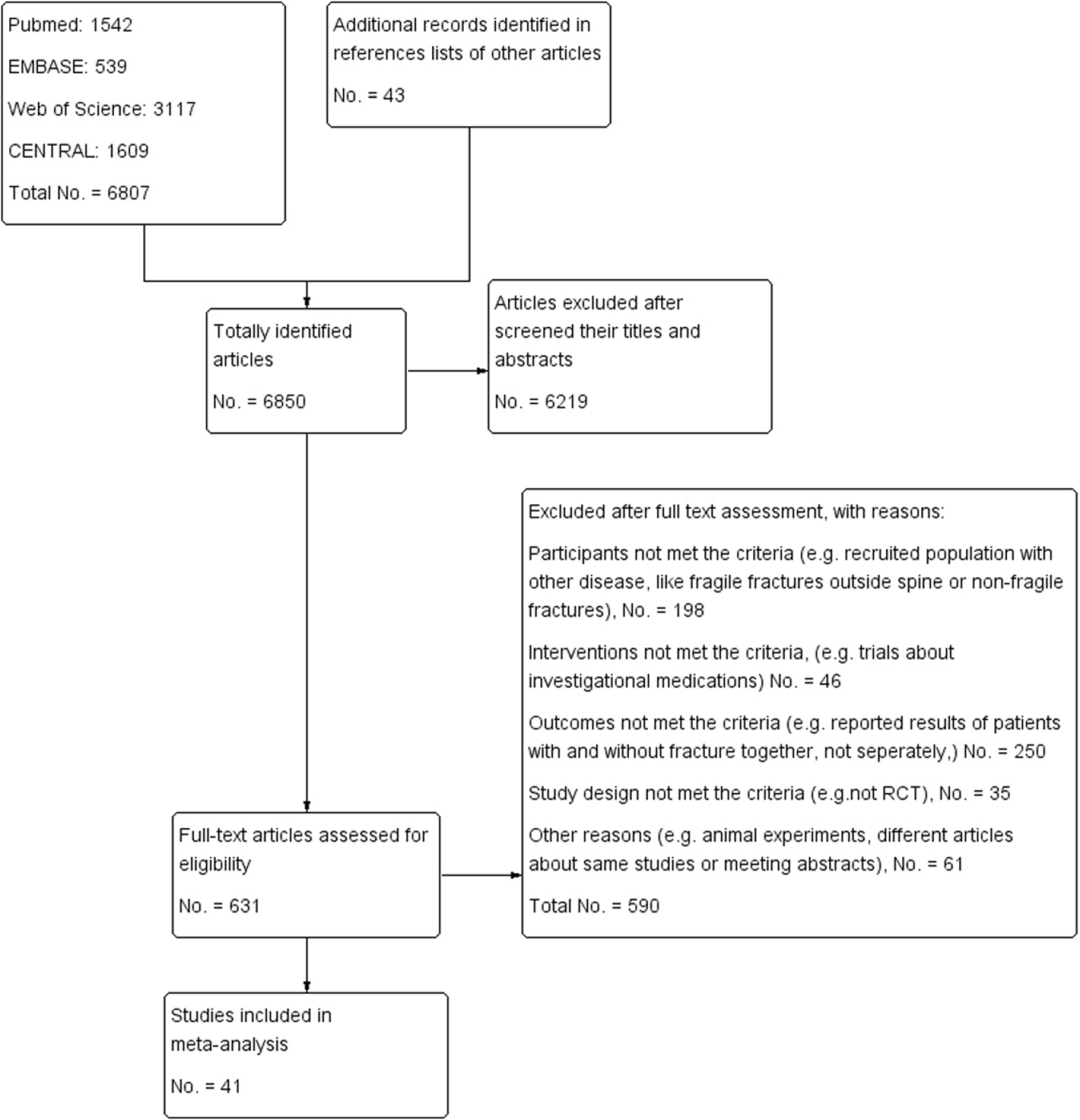
Flow chart of selected studies

A total of 6850 articles were identified. Among them, 631 were subjected to full-text assessment. After full-text examination, 41 articles were finally included in this study (Fig. 1). Among them, 34 compared the effects of medications with control groups. Bisphosphonates (BPs) were compared in 19 RCTs [17–33, 35, 36], calcitonin in 3 [37–39], hormone replacement therapy (HRT) in 3 [40–42], parathyroid hormone (PTH), teriparatide, or abaloparatide in 5 [43–47], denosumab in 2 [48, 49], and selective estrogen receptor modulators (SERMs) in 3 [50–52]. Five trials compared between the effects of medications, risedronate vs. etidronate [53], ibandronate vs. risedronate [54], romosozumab vs. alendronate [55], and teriparatide vs. risedronate [57, 58]. Follow-up duration in most trials was 2 to 3 years. Other basic characteristics of included studies were summarized in Table 1.

**Table 1:** Characteristics of included studies

Study ID	Number of participants had prevalent fractures	Proportion of participants had prevalent fractures	Mean age (year)	Intervention & Comparison	Calcium	Vitamin D	Observation period (year)	Lost to follow up
Compare with control group								
*Zoledronate*								
Nakamura, 2017 [17]	661	100%	74.15	G1: Zoledronate 5 mg/year, intravenous infusion; G2: PLC	Both groups	Both groups	2	0.6%
*Alendronate*								
Black, 1996 [18]	1942	100%	71	G1: Alendronate 5 mg/d on the first 2 years, 10 mg/d on the third year G2: PLC	Selectively offer	Selectively offer	3	9%
Kushida, 2004 ^a^ [19]	170	100%	72	G1: Alendronate 5 mg/d G2: Alfacalcidol 1 μg/d	1.5 g/d	Alfacalcidol	3	30%
Liberman, 1995 ^a^ [20]	165	18.72%	64	G1: Alendronate 5–10 mg/d G2: PLC	All groups	Not reported	3	16%
*Risedronate*								
Clemmesen, 1997 [21]	132	100%	68	G1: Risedronate 2.5 mg/d continuously G2: Risedronate 2.5 mg/ cyclically G3: PLC	All groups	Not reported	3	30%
Reginster, 2000 [22]	690	100%	71	G1: Risedronate 5 mg/d; G2: Risedronate 2.5 mg/d; G3: PLC	All groups	All groups	3	42%
Sorensen, 2003 [23]	212	100%	72	G1: Risedronate 5 mg/d G2: PLC	Both groups	Both groups	2	17%
Fogelman, 2000 ^a^ [24]	237	43.81%	64	G1: Risedronate 2.5 mg/d G2: Risedronate 5 mg/d G3: PLC	All groups	Not reported	2	21%
Harris, 1999 [25]	1374	100%	69	G1: Risedronate 5 mg/d; G2: Risedronate 2.5 mg/d; G3: PLC	All groups	All groups	3	42%
*Etidronate*								
Guanabens, 2000 [26]	118	100%	65	G1: Etidronate 400 mg/d for 14 days in a cyclic of 90 days G2: Sodium fluoride 50 mg/d	Selectively offer	Not reported	3	34%
Lyritis, 1997 [27]	100	100%	72	G1: Etidronate 400 mg/d for 20 days in a cyclic of 90 days G2: 5 days’ vitamin D + 85 days calcium	Both groups	Both groups	4	26%
Montessori, 1997 ^a^ [28]	28	35%	62.5	G1: Etidronate 400 mg/d for 14 days in a cyclic of 90 days G2: Calcium 500 mg/d	Selectively offer	Not reported	3	20%
Shiota, 2001 ^a^ [29]	24	60%	61.7	G1: Etidronate 200 mg/d for 14 days in a cyclic of 84 days G2:2 g/d calcium and 0.5 μg/d alphacalcidol for 2 years	Selectively offer	Selectively offer	2	Not reported
Harris, 1993 [36]	423	100%		G1: PLC and PLC G2: Phosphate and PLC G3: PLC and Etidronate 400 mg/daily for 14 in a cycle of 91 days G4: Phosphate and Etidronate	All groups	Not mentioned	4^c^	20%
Watts, 1990 [30]	423	100%	65	G1: PLC for 17 days in a cyclic of 91 days G2: Phosphonate 2 g/d for 3 days in a cyclic of 91 days G3: Etidronate 400 mg/d for 14 days in a cyclic of 91 days G4: Phosphonate 2 g/d for 3 days + Etidronate 400 mg/d for next 14 days in a cyclic of 91 days	All groups	Not reported	2	14%
*Ibandronate*								
Chesnut, 2004 [31]	2929	100%	69	G1: Ibandronate 2.5 mg/d, oral G2: Ibandronate 20 mg alternate day for 12 doses every 3 months, oral G3: PLC	All arms	All arms	3	34%
Recker, 2004 [32]	2860	100%	67	G1: Ibandronate 0.5 mg injection, every 3 months G2: Ibandronate 1 mg injection, every 3 months G2: PLC	All arms	All arms	3	18%
*Minodronate*								
Matsumoto, 2009 [33]	704	100%	72	G1: Minodronate 1 mg/d G2: PLC	Both groups	Both groups	2	31%
*Pamidronate*								
Reid, 1994 [34]	61	100%	66	G1: Pamidronate 150 mg/d G2: PLC	Both groups	Not reported	2	79%
Brumsen, 2002 [35]	101	100%	65	G1: Pamidronate 150 mg/d G2: PLC	Both groups	Both groups	3	10%
*Calcitonin*								
Peichl, 1999 [37]	42	100%	62	G1: Nasal salmon calcitonin 100 IU twice daily for 2 months with a pause of 2 months G2: Control group	Both groups	Only control groups	1	Not reported
Hodsman, 1997 [38]	30	100%	67	G1: PTH sc injections 800 IU/d for 28 days in a cyclic of 90 days G2: PTH sc injections 800 IU/d for 28 days + salmon calcitonin 75 U/d for 42 days in a cyclic of 90 days	Both groups	Not prescribed	2	23%
Chesnut, 2005 [39]	91	100%	67.4	G1: Calcitonin nasal spray 200 IU/d G2: Placebo nasal spray	Both groups	Not reported	2	78%
*Hormone replace therapy*								
Gutteridge, 2002 [40]	99	100%	69	G1: Fluoride G2: Control group G3: Fluoride + Estrogen 0.625 mg/d G4: Estrogen 0.625 mg/d	All groups	All groups	2.25	24%
Wimalawansa, 1998 [41]	72	100%	65	G1: HRT group, Permarin 0.625 mg/d + norgestril 150 μg for 12 days each month G2: Etidronate group, Etidronate 400 mg/d for 14 days each 12 week G3: Combined therapy, combination of G1 and G2 with same dose G4: control group	All groups	All groups	4	17%
Lufkin, 1992 [42]	75	100%	65	G1: Estrogen group, Estradiol 0.1 mg/d on the first 21 days + medroxyprogesterone acetate for the days 11 to 21 in a 28 days’ cycle G2: Placebo	Both	Not reported	1	
*Parathyroid hormone*								
Neer, 2001 [43]	1637	100%	71.0	G1: rhPTH 20 μg/d G2: rhPTH 40 μg/d G3: PLC	All groups	All groups	2	6%
Nakamura, 2012 [44]	578	100%	75.3	G1: Teriparatide 56.5 μg/w, sc injection G2: PLC, sc injection	Both groups	Both groups 400 IU/d	1.5	26%
Greenspan, 2001^a^ [46]	471	18.6%	64.4	G1: Teriparatide 100 μg/d, sc injection G2: PLC	Both groups	Both groups	1.5	33%
Fujita, 2014 [45]	316	100%	71	G1: Teriparatide 28.2 μg/w, injection G2: Teriparatide 1.4 μg/w, injection	Both groups	Not prescribed	3	17%
*Denosumab*								
Nakamura, 2014 [48]	1262	100%	69.6	G1: Denosumab 60 mg/6 months, sc injection G2: PLC G3: Alendronate 35 mg/w	All groups	All groups	3	13%
Boonen, 2011 ^b^ [49]	759	100%	73.7	G1: Denosumab 60 mg/6 months, sc G2: PLC	Both groups	Both groups	3	18%
*Romozumab*								
Saag, 2017 [55]	4093	100%	74.3	G1: Alendronate: 70 mg/w G2: Romosozumab: 210 mg/m sc injection	Both groups	Both groups	3	11%
*Raloxifene*								
Ettinger, 1999 ^a^ [50]	2304	33.74%	68	G1: Raloxifene 60 mg/d G2: Raloxifene 120 mg/d G3: PLC	All groups	All groups	3	23%
Lufkin, 1998 [51]	143	100%	68	G1: Raloxifene 60 mg/d G2: Raloxifene 120 mg/d G3: PLC	All groups	All groups	1	9%
*Bazedoxifene*								
Palacios, 2015 ^a^ [52]	3857	49.40%	67	G1: Bazedoxifene 60 mg/d G2: Bazedoxifene 40 mg/d G3: Bazedoxifene 20 mg/d G4: PLC	All groups	All groups	7	74%
Compare between medications								
Kushida, 2004 b [53]	547	100%	72	G1: Risedronate 2.5 mg/d G2: Etidronate 200 mg/d cyclically	Both groups	Not reported	2	21%
Nakamura, 2013 [56]	1265	100%	72.7	G1: Ibandronate 0.5 mg injection per month G2: Ibandronate 1 mg iv injection per month G3: Risedronate 2.5 mg/d	All arms	All arms	3	10%
Hadji, 2012 [57]	710	100%	71	G1: Risedronate: 35 mg/w G2: Teriparatide: 20 μg/w subcutaneous injection	Both groups	Both groups	1.5	26%
Kendler, 2017 [58]	1360	100%	72.1	G1: Risedronate 35 mg/w G2: Teriparatite: 20 μg/d subcutaneous injection	Both groups	Both groups	2	26%

*PLC* Placebo, *PTH* Parathyroid hormone, *ALD* Alendronate

^a^The study included patients with and without fracture history, and only the data of patients with fracture history was analyzed

^b^Post hoc analysis of previous data

^c^Data on the third year was pooled because the study design was changed to open-label and all participants in the fourth year received Etidronate

Approximately half of the biases were rated as unclear risk (Additional file 2). Risk of other sources of bias was rated as high in one study because the criteria used in its two clinical centers were different [21]. Performance bias was rated as high risk in 6 trials for significantly different compliance between groups [26, 46, 52] and the open-label study design used in 4 trials [20, 27, 37, 40].

Fujita et al. treated a teriparatide 1.4 μg/week group as a placebo group, and therefore, we followed their grouping and classified their data of teriparatide 1.2 μg/week group as the control group [45]. On the other side, since the control groups in other studies all received placebo, which was different from Fujita et al.’s study, it might affect the final result. Therefore, we performed a sensitivity analysis about this result, with excluding the Fujita el al.’s study from the original analysis and then compared the results from original analysis and sensitivity analysis (Table 1). Sorensen et al. reported a 2-year extension trial [23] of a 3-year original trial [22]. In the entension trial, the authors treated the initial time point of the extention trial as baseline. Therefore, while synthesizing the data, we deemed the data from the two studies were not duplicated and synthesized the data as from two studies. However, because the participants in experimental group and control group in the extension study had different medication history, the risk of selection bias of the extended trial was rated as high (Additional file 2).

### Comparison with control group

#### Antiresorptive medications

The result of antiresorptive medications, including BPs, HRT, SERMs, calcitonin, and denosumab, were pooled together to investigate the effects of the medications. Thirty-three studies involving 21,012 participants were included. The result indicated that the administration of antiresorptive medications could significantly reduce the risk of the secondary OVCF (RR, 0.59; 95% CI, 0.53–0.65, *p* < 0.00001) (Table 2, Additional file 3a). Bisphosphonates did not significantly increase gastrointestinal (GI) complaints (RR, 1.02, *p* = 0.45; Additional file 3b). The result was treated as a secondary outcome because the heterogeneity in the comparison.

**Table 2:** Summary of findings of osteoporotic vertebral fracture and non-vertebral fracture

Comparison	RR (95% CI)	No. of participants (studies)	Quality of the evidence
Vertebral fracture			
Antiresorptive medication vs. Control	0.59 (0.53 to 0.65)	21,012 (30 RCTs)	–
ZOL vs. Control	0.34 (0.17 to 0.69)	657 (1 RCT) ^a^	MODERATE ^b,^
ALN vs. Control	0.54 (0.43 to 0.68)	2277 (3 RCTs) ^c^	HIGH
RISE vs. Control	0.61 (0.51 to 0.73)	2645 (5 RCTs) ^d^	MODERATE ^e^
Etidronate vs. Control	0.60 (0.39 to 0.92)	618 (7 RCTs) ^f^	MODERATE ^g,^
Ibandronate (sufficient) vs. Control	0.52 (0.38 to 0.71)	2929 (1 RCT) ^h^	MODERATE ^i^,
Ibandronate (insufficient) vs. Control	0.87 (0.69 to 1.11)	2860 (1 RCT) ^j^	MODERATE ^k^
Minodronate vs. Control	0.44 (0.31 to 0.63)	674 (1 RCT) ^l^	LOW ^m^
Pamidronate vs. Control	0.33 (0.13 to 0.84)	90 (1 RCT) ^n^	VERY LOW ^o^
Calcitonin vs. Control	1.02 (0.14 to 7.36)	157 (3 RCTs) ^p^	VERY LOW ^q^
HRT vs. Control	0.86 (0.29 to 2.52)	147 (3 RCTs) ^r^	LOW ^s^
PTH vs. Control	0.32 (0.24 to 0.43)	2632 (4 RCTs) ^t^	MODERATE ^u^
Denosumab vs. Control	0.41 (0.29 to 0.57)	1654 (2 RCTs) ^v^	MODERATE ^w^
RLX vs. Control	0.58 (0.44 to 0.76)	2447 (2 RCTs) ^x^	HIGH
BZA vs. Control	0.66 (0.53 to 0.82)	3857 (1 RCT) ^y^	MODERATE ^z^
ALN vs. Denosumab	0.69 (0.41 to 1.17)	722 (1 RCT) ^aa^	LOW ^bb^
Romosozumab vs. Alendronate	0.64 (0.49 to 0.84)	4093 (1 RCT)^cc^	MODERATE^dd^
RISE vs. Etidronate	1.12 (0.69 to 1.81)	433 (1 RCT) ^ee^	MODERATE ^ff^
Ibandronate vs. RISE	1.01 (0.79 to 1.31)	1228 (1 RCT)^gg^	HIGH
RISE vs. Teriparatide	1.98 (1.44 to 2.70)	2070 (2 RCTs) ^hh^	HIGH
HRT vs. Etidronate	0.63 (0.12 to 3.32)	35 (1 RCT) ^ii^	VERY LOW ^jj^
Non-vertebral fracture			
ZOL vs. Control	0.54 (0.32 to 0.91)	661 (1 RCT)^kk^	–
ALN vs. Control	0.81 (0.65 to 1.01)	2027 (1 RCT)^ll^	–
RISE vs. Control	0.71 (0.54 to 0.92)	2836 (4 RCTs)^mm^	–
Etidronate vs. Control	0.95 (0.59 to 1.53)	395 (4 RCTs)^nn^	–
Ibandronate (sufficient) vs. Control	1.10 (0.85 to 1.41)	2929 (1 RCT)^oo^	–
Ibandronate (insufficient) vs. Control (only Hip fracture)	0.59 (0.26 to 1.31)	2860 (1 RCT)^pp^	–
Minodronate vs. Control	0.80 (0.35 to 1.84)	674 (1 RCT)^qq^	–
Pamidronate vs. Control	0.33 (0.04 to 3.10)	100 (1 RCT)^rr^	–
PTH vs. Control	0.53 (0.36 to 0.78)	2454 (3 RCTs)^ss^	–
Denosumab vs. Control	0.45 (0.20 to 1.03)	952 (1 RCT)^tt^	–
Romosozumab vs. ALN	0.74 (0.54 to 1.00)	4093 (1 RCT)^uu^	
ALN vs. Dmab	1.49 (0.52 to 4.24)	722 (1 RCT)^vv^	–
Ibandronate vs. RISE	1.12 (0.75 to 1.66)	1134 (1 RCT)^gg^	–
RISE vs. Teriparatide	1.28 (0.94 to 1.73)	2070 (2 RCTs)^ww^	–
HRT vs. Etidronate	0.94 (0.06 to 13.93)	35 (1 RCT)^xx^	–

*RR* Relative Risk, *ZOL* Zoledronate, *ALN* Alendronate, *RISE* Risedronate, *PTH* Pamidronate, *RLX* Raloxifene, *BZA* Bazedoxifene, *HRT* Hormone replace therapy

^a^Nakamura, 2017 [17]

^b^Study limitations: the trial included had unclear risk of performance bias

^c^Liberman, 1995 [20]; Black, 1996 [18]; Kushida, 2004 [19, 53]

^d^Clemmesen, 1997 [21]; Harris, 1999 [25]; Reginster, 2000 [22]; Fogelman, 2000 [24]; Sorensen, 2003 [23]

^e^Study limitations: four trials were included, with unclear risk of selection bias, performance bias and attribution bias

^f^Shiota, 2001 [29]; Montessori, 1997 [28]; Lyritis, 1997 [27]; Watts, 1990 [30]; Harris, 1993 [36]; Wimalawansa, 1998 [41]; Guanabens, 2000 [26]

^g^Study limitations: seven trials were included, with unclear to high risk of selection bias, attribution bias, other bias, and performance bias

^h^Chesnut, 2004 [31]

^i^Study limitations: one trial was included, with unclear risk of performance bias and attribution bias

^j^Recker, 2004 Recker, 2004 [32]

^k^One trial included, with unclear risk of performance bias and other bias

^l^Matsumoto, 2009 [33]

^m^One trial was included, with unclear risk of performance bias, attribution bias and other source of bias. Imprecision: the number of events was 115 and OIS was not met

^n^Brumsen, 2002 [35]

^o^Study limitation: one trial included, with unclear risk of selection bias. Imprecision (rating down two levels): 20 events and CIs included appreciable benefit

^p^Hodsman, 1997 [38]; Peichl, 1999 [37]; Chesnut, 2005 [39]

^q^Study limitation: two trials had unclear to high risk of selection bias, performance bias, attribution bias and other bias. Imprecision (rating down two levels): 15 events and CIs included appreciable benefit and harm

^r^Lufkin, 1992 [42]; Wimalawansa, 1998 [41]; Gutteridge, 2002 [40]

^s^Study limitation: two trials had unclear risk of selection bias. Two trials had unclear to high risk of performance bias. Three trials had unclear risk of attribution bias. Three trials had unclear risk of other bias. Imprecision (rating down two levels): 34 events and CIs included appreciable benefit and harm

^t^Nakamura, 2012 [44], Neer, 2001 [43], Greenspan, 2007, Fujita, 2014 [45]

^u^One trial had unclear risk of selection bias, performance bias and attribution bias. One trial had high risk of performance bias

^v^Boonen, 2011 [49]; Nakamura, 2014 [48]

^w^Study limitation: two trials had unclear risk of selection bias and performance bias. One trial had had unclear risk of other bias

^x^Ettinger, 1999 [50], Lufkin, 1998 [51]

^y^Palacios, 2015 [52]

^z^Study limitation: one trial had high risk of performance bias and unclear risk of attribution bias and other bias

^aa^Nakamura, 2014 [48]

^bb^Study limitation: one study included, with unclear risk of selection bias, performance bias, and other bias. Imprecision: the number of events was 66, and OIS was not met

^cc^Saag, 2017 [55]

^dd^Study limitation: one trial was included, with unclear risk of performance bias

^ee^Kushida, 2004 [53]

^ff^Study limitation: one trial was included, with unclear risk of selection bias, attribution bias and other bias

^gg^Nakamura, 2013

^hh^Hadji, 2012 [57]; Kendler, 2017 [58]

^ii^Wimalawansa, 1998 [41]

^jj^Study limitation: one trial was included, with unclear risk of performance bias, attribution bias and other bias. Imprecision (rating down two levels): few events and CIs included appreciable benefit and harm

^kk^Nakamura, 2017 [17]

^ll^Black, 1996 [18]

^mm^Clemmesen, 1997 [21]; Harris, 1999 [25]; Reginster, 2000 [22]; Sorensen, 2003 [23]

^nn^Watts, 1990 [30]; Lyritis, 1997 [27]; Montessori, 1997 [28]; Guanabens, 2000 [26]

^oo^Chesnut, 2004 [31]

^pp^Recker, 2004 [32]

^qq^Matsumoto, 2009 [33]

^rr^Brumsen, 2002 [35]

^ss^Nakamura, 2012 [44]; Neer, 2001 [43], Fujita, 2014 [45]

^tt^Nakamura, 2014 [48]

^uu^Saag, 2017 [55]

^vv^Nakamura, 2014 [48]

^ww^Hadji, 2012 [57]; Kendler, 2017 [58]

^xx^Wimalawansa, 1998 [41]

#### Zoledronate

**Fig. 2:**
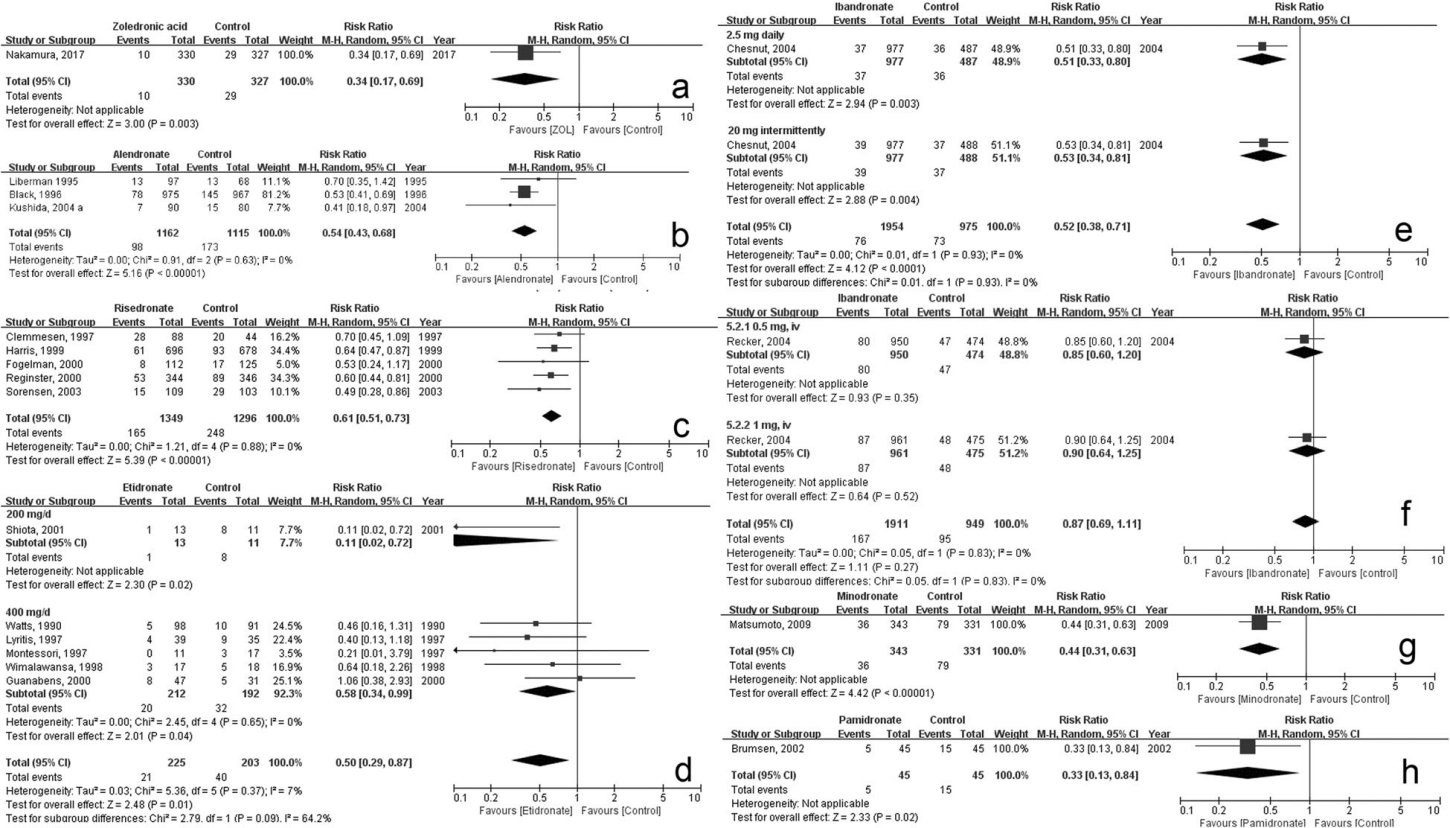
Forest plot of the secondary prevention effects of bisphosphonates. **a** Zoledronate; **b** Alendronate; **c** Risedronate; **d** Etidronate; **e** Ibandronate (sufficient dose); **f** Ibandronate (insufficient dose); **g** Minodronate; **h** Pamidronate

Moderate quality evidence proved that zoledronate could significantly decrease the risk of secondary OVCF (RR, 0.34; 95% CI, 0.17–0.69, *p* = 0.003; Fig. 2a, Table 2), without significant increase in discontinuation due to medication (RR, 1.99; 95% CI, 0.76–5.25, *p* = 0.16; Table 3, Additional file 3c). Additionally, zoledronate could significantly decrease event ratio of non-vertebral fractures (RR, 0.54; 95% CI, 0.32–0.91; *p* = 0.02; Table 2, Additional file 3d).

**Table 3:** Discontinuation due to medication

Comparison	No. of participants (studies)	RR (95% CI)
ZOL vs. Control	665 (1 RCT)	1.99 (0.76, 5.25)
ALN vs. Control	2750 (2 RCTs)	0.88 (0.64, 1.22)
RISE vs. Control	2707 (3 RCTs)	0.88 (0.69, 1.12)
Etidronate vs. Control	322 (3 RCTs)	0.40 (0.03, 5.48)
Ibandronate vs. Control		
2.5 mg/d & 20 mg alternatively	2929 (1 RCT)	0.90 (0.69, 1.18)
0.5 mg & 1 mg per 3 months	2860 (1 RCT)	1.27 (0.97, 1.66)
PTH vs. Control	2215 (2 RCTs)	1.54 (1.11, 2.13)
56.5 μg/w	578 (1 RCT)	1.80 (1.00, 3.24)
20 μg/d	813 (1 RCT)	1.10 (0.62, 1.95)
40 μg/d	824 (1 RCT)	1.82 (1.07, 3.10)
Denosumab vs. Control	956 (1 RCT)	0.75 (0.44, 1.27)
HRT vs. Control	79 (2 RCTs)	0.53 (0.17, 1.61)
ALN vs. Denosumab	717 (1 RCT)	0.79 (0.15, 4.02)
Romosozumab vs. Alendronate	4093 (1 RCT)	1.00 (0.58, 1.74)
RISE vs. Teriparatide	2070 (2 RCT)	0.75 (0.57, 1.00)
HRT vs. Etidronate	35 (1 RCT)	2.83 (0.33, 24.66)

*RR* Relative Risk, *ZOL* Zoledronate, *ALN* Alendronate, *RISE* Risedronate, *PTH* Pamidronate, *HRT* Hormone replace therapy

#### Alendronate

High quality evidence proved that administrating alendronate significantly reduced the proportion of participants who had subsequent vertebral fractures (RR, 0.54; 95% CI, 0.43–0.68; *p* < 0.0001; heterogeneity, *p* = 0.63, I^2^ = 0%; Fig. 2b, Table 2). No significant increase in GI complaints or discontinuation was observed in the alendronate group (GI complaints, RR, 1.03; 95% CI, 0.93–1.15, *p* = 0.55; Discontinuation, RR, 0.88; 95% CI, 0.64–1.22, *p* = 0.46; Table 3, Additional file 3e and f). Alendronate had no significant effect on preventing non-vertebral fractures (RR, 0.81; 95% CI, 0.65–1.01, *p* = 0.07; Table 2, Additional file 3g).

#### Risedronate

Moderate quality evidence indicated that risedronate had a significant effect on preventing subsequent vertebral fractures (RR, 0.61; 95% CI, 0.51–0.73, *p* < 0.0001; Fig. 2c, Table 2). Risedronate administration did not significantly elevate GI complaints (RR, 1.09; 95% CI, 0.96–1.23, *p* = 0.18) or discontinuation rate (RR, 0.88; 95% CI, 0.69–1.12, *p* = 0.28) (Table 3, Additional file 3h and i). Risedronate had a significant effect on preventing non-vertebral fractures (RR, 0.71; 95% CI, 0.54–0.92, *p* = 0.01; Table 2, Additional file 3j).

#### Etidronate

Moderate quality evidence showed that the administration of etidronate could significantly reduce the risk of subsequent vertebral fractures (RR, 0.50; 95% CI, 0.29–0.87, *p* < 0.01; Fig. 2d, Table 2). The result was consistent with that of sensitivity test, in which a study [29] with a small sample size and big variance was excluded (Additional file 3k). No significant difference was observed in GI complaints (RR, 0.57; 95% CI, 0.28–1.15, *p* = 0.12) or discontinuation (RR, 0.40; 95% CI, 0.03–5.48, *p* = 0.50) between intervention and control groups (Table 3, Additional file 3l and m). Etidronate did not have a significant effect on preventing non-vertebral fractures (RR, 0.95; 95% CI, 0.59–1.53, *p* = 0.83; Table 2, Additional file 3n).

#### Ibandronate

Moderate quality evidence proved that ibandronate administrated 2.5 mg daily or 20 mg intermittently could significantly reduce the subsequent fracture risk (RR, 0.52; 95% CI, 0.38–0.71, *p* < 0.0001; Fig. 2e, Table 2), while insufficient dosages (0.5 mg or 1 mg per 3 months) did not (RR, 0.87; 95% CI, 0.69–1.11, *p* = 0.27; Fig. 2f, Table 2). Ibandronate did not significantly raise the risk of discontinuation due to adverse events (sufficient dose: RR, 0.90; 95% CI, 0.69–1.18, *p* = 0.45; insufficient dose: RR, 1.27; 95% CI, 0.98–1.66, *p* = 0.07) (Additional file 3o and p, Table 3). Neither sufficient nor insufficient dosage of ibandronate had significant effect on preventing non-vertebral fractures (sufficient: RR, 1.10; 95% CI, 0.85–1.41, *p* = 0.47; insufficient, only hip fracture: RR, 0.59; 95% CI, 0.26–1.31; *p* = 0.19; Table 2, Additional file 3q and r).

#### Minodronate

Low quality evidence proved minodronate had significant effect in reducing secondary fracture (RR, 0.44; 95% CI, 0.31–0.63; *p* < 0.001; Fig. 2g, Table 2). Minodronate did not have a significant effect on preventing non-vertebral fractures (RR, 0.80; 95% CI, 0.35–1.84, *p* = 0.60; Table 2, Additional file 3s).

#### Pamidronate

Very low quality evidence indicated significantly lower risk of secondary fracture due to pamidronate (RR, 0.33; 95% CI, 0.13–0.84, *p* = 0.02; Fig. 2h, Table 2). Pamidronate did not have significant effect on preventing non-vertebral fractures (RR, 0.33; 95% CI, 0.04–3.10, *p* = 0.33; Table 2, Additional file 3t).

#### Calcitonin

Very low quality evidence proved calcitonin had no significant effect on preventing secondary fracture (RR, 1.02; 95% CI, 0.14–7.36, *p* = 0.98) (Table 2).

#### HRT

Low quality evidence proved HRT had no significant effect on prevention of secondary vertebral or non-vertebral fracture (vertebral: RR, 0.88; *p* = 0.78; non-vertebral: RR, 0.37; 95% CI, 0.04–3.05, *p* = 0.36; Table 2, Additional file 3u). HRT did not significantly elevate the risk of discontinuation (RR, 0.53, 95% CI, 0.17–1.61, *p* = 0.26; Table 3, Additional file 3v).

#### Parathyroid (PTH)

**Fig. 3:**
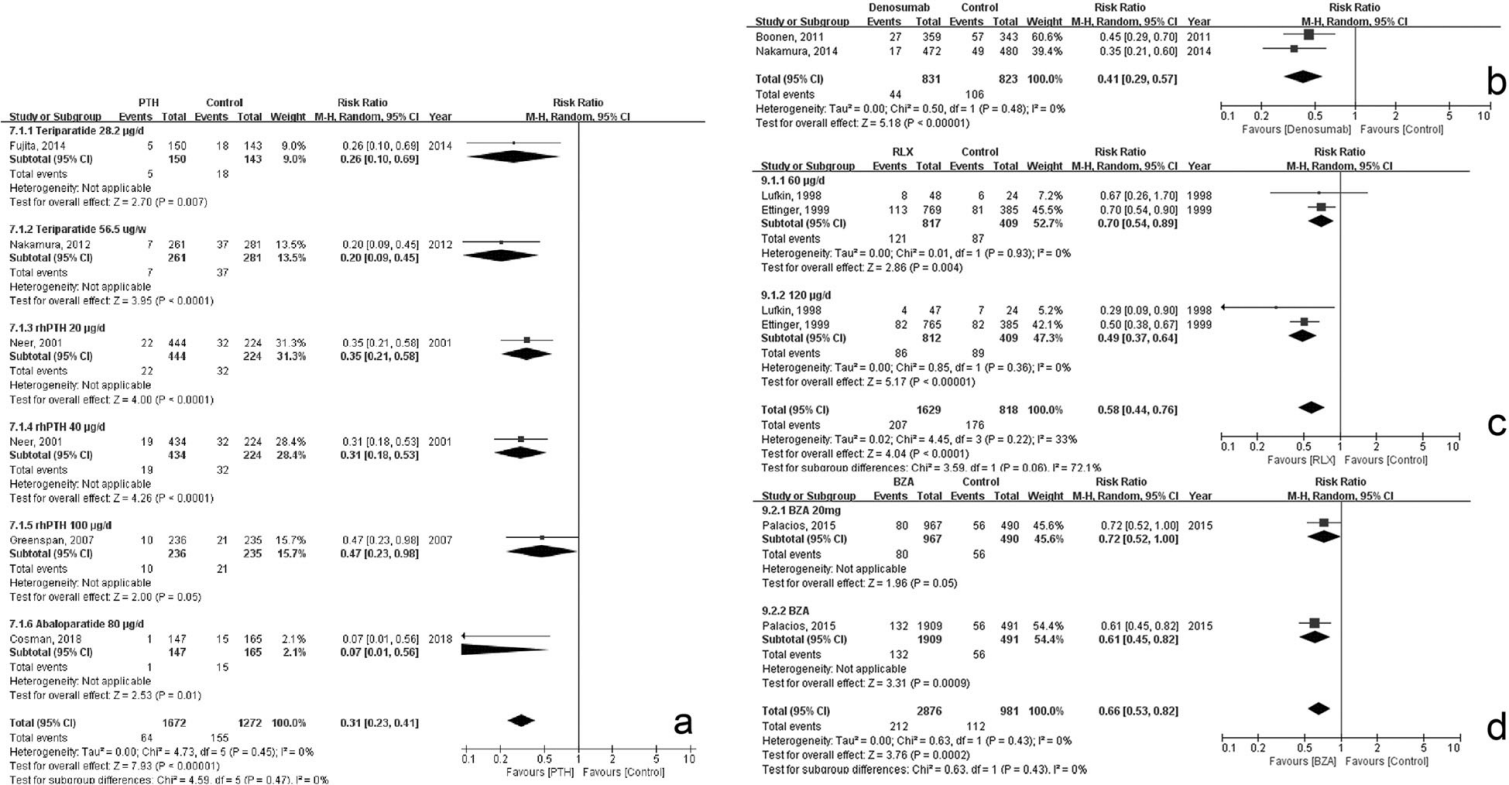
Forest plot of the secondary prevention effects of: **a** Parathyroid; **b** Denosumab; **c** Raloxifene; and **d** Bazedoxifene

Moderate quality evidence proved that the administration of teriparatide 28.2 μg/week or 56.5 μg/week, abaloparatide 80 μg/day, and recombinant human (rh)PTH 20 μg/day or rhPTH 40 μg/day could significantly reduce the risk of secondary fracture (Table 2). The synthesized RR was 0.31 (95% CI, 0.23–0.41; *p* < 0.0001) and the heterogeneity between different doses was insignificant (*p* = 0.45, Fig. 3a). The result of the sensitive analysis that excluded the trial had teriparatide 1.4 μg/week group as its control group [45] showed no significant change (RR, 0.31, 95% CI, 0.22–0.44, *p* < 0.00001). The risk of discontinuation due to medication was significantly raised by PTH administration (RR, 1.54; 95% CI, 1.11–2.13, *p* < 0.009; Additional file 3w). Forty μg/day rhPTH significantly elevated the risk of discontinuation, while 20 μg/day or 56.5 μg/week did not, but no significant heterogeneity was observed between groups. PTH had significant effect on preventing non-vertebral fractures (RR, 0.52; 95% CI, 0.36–0.75; *p* = 0.0005; Table 2, Additional file 3x).

#### Denosumab

Moderate quality evidence proved that the administration of denosumab significantly reduced the risk of secondary fracture (RR, 0.41; 95% CI, 0.29–0.57; *p* < 0.0001; Fig. 3b, Table 2). No significant increase in discontinuation due to medication was observed (RR, 0.75; 95% CI, 0.44–1.27, *p* = 0.29; Table 3, Additional file 3y). Denosumab did not have a significant effect on preventing non-vertebral fractures (RR, 0.45; 95% CI, 0.20–1.03, *p* = 0.06; Table 2, Additional file 3z).

#### SERMs

Both raloxifene (RLX) and bazedoxifene (BZA) could significantly reduce risk of secondary fracture (RLX: RR, 0.58; 95% CI, 0.44–0.76, *p* < 0.0001. BZA: RR, 0.66; 95%CI, 0.53–0.82, *p* = 0.0002; Fig. 3c and d). Heterogeneity between 60 μg/day and 120 μg/day of RLX was significant and substantial (test for subgroup differences, *p* = 0.06, I^2^ = 72.1%; Fig. 3c). The effect of BZA was proved by moderate quality evidence and the effect of RLX was supported by high quality evidence (Table 2).

### Comparison between interventions

#### Comparison between BPs

Moderate quality evidence proved no significant difference in the effects on preventing vertebral fracture between risedronate and etidronate (RR, 1.12; 95% CI, 0.69–1.81, *p* = 0.66; Additional file 4a). High quality evidence proved no significant difference between ibandronate and risedronate in preventing vertebral fracture (RR, 1.01; 95% CI, 0.78–1.32, *p* = 0.92; Additional file 4b, Table 2) and no significant difference was observed between ibandronate and risedronate in preventing non-vertebral fracture (RR, 1.12; 95% CI, 0.75–1.66, *p* = 0.59; Additional file 3aa, Table 2).

#### Hormone therapy vs. BPs

Very low quality evidence indicated no significant difference between HRT and etidronate (RR, 0.63; 95% CI, 0.12–3.32, *p* = 0.59; Additional file 4c). Moderate quality evidence indicated teriparatide (20 μg/week) showed a significantly superior effect on preventing vertebral fracture and non-vertebral fracture than risedronate (vertebral fracture: RR, 1.98; 95% CI, 1.44–2.7, *p* < 0.0001; Additional file 4d), without significantly increasing ratio of discontinuation (RR, 0.75; 95% CI, 0.57–1.00, *p* = 0.05; Table 3, Additional file 3bb). No significant difference in effects of non-vertebral fracture was observed (RR, 1.28; 95% CI, 0.94–1.73, *p* = 0.12; Additional file 3cc).

#### Monoclonal antibody medication vs. BPs

Low quality evidence proved the difference between the effects of alendronate and denosumab on preventing vertebral fracture was not significant (RR, 0.69; 95% CI, 0.41–1.17, *p* = 0.17; Additional file 4e). Moderate quality evidence proved romosozumab had significantly better effect on preventing secondary vertebral fracture than alendronate (RR, 0.64, 95% CI, 0.49–0.84, *p* = 0.001; Additional file 4f).

Difference between the effects of alendronate and denosumab on preventing non-vertebral fracture was not statistically different (RR, 1.49; 95% CI, 0.52–4.24; *p* = 0.46; Additional file 3dd), neither was between romosozumab and alendronate (RR, 0.74; 95% CI, 0.54–1.00, *p* = 0.05; Additional file 3ee).

## Discussion

In this study, we focused on osteoporosis patients with a history of OVCF. We collected related RCTs, synthesized their results, and finally estimated the secondary prevention effects of the medications on OVCF. We found zoledronate, alendronate, risedronate, etidronate, ibandronate, minodronate, pamidronate, PTH, denosumab, romosozumab and SERMs had significant secondary prevention effect on OVCF. In the comparisons between the medications, teriparatide had a significantly superior effect to risedronate, and the quality of evidence was high. The effects of risedronate, ibandronate, PTH, and SERMs were supported by moderate quality evidence and the effects of alendronate, denosumab were supported by high quality evidence.

In the result of discontinuation due to adverse events, PTH was the only intervention that significantly elevated the ratio. None of the bisphosphonates increased the risk of GI complaints. Zoledronate, risedronate, and PTH had significant effect on preventing non-vertebral fracture in patients with prevalent OVCF.

Most of widely used BPs, include zoledronate, alendronate, risedronate, etidronate and ibandronate, had significant effect, which were supported by moderate quality evidences. Among the medications, risedronate and ALN are first line osteoporosis medications, whose effects have been proved by substantial evidence [5, 7]. Ibandronate is a nitrogen-containing BPs and IV injection of it allows for a dosing interval even longer than 2 months [59]. Zoledronate is another nitrogen-containing BPs that has the highest potency among clinical use BPs [60]. According to our result, 5 mg/year iv injection of zoledronate could significantly reduce the risk of secondary OVCF. The extremely low medication frequency could be its another advantage that might improve patients’ compliance rate. Significantly elevated adverse events ratio or rare adverse events caused by BPs (e.g. osteonecrosis of jaw or atypical fracture, etc.) was not 7reported in any trial. Insignificant difference in GI complaints between BPs and control group indicated properly administrated BPs might help avoiding the risk of GI complaints, which was consistent with previous studies [61].

PTH is a bone anabolic medication that has significant efficacy against OVCF [62]. In this study, moderate quality evidence proved that the injection of PTH or teriparatide significantly reduce the risk of secondary OVCF and even the lowest dosage (28.2 μg/week) showed a significant effect. Compare with risedronate, teriparatide showed significantly better effect, which indicated PTH might have better effect on preventing secondary OVCF. It was consistent with previous studies that proved PTH had better effect on spine BMD compare with bisphosphonate [63, 64]. But on the other side, the superiority of PTH over bisphosphonate on hip BMD remains controversial, and it has been showed the PTH had inferior effect on BMD of distal radius [63, 64]. .Also, PTH treatment was the only medication that was associated with a series of adverse events that increased the risk of discontinuation. The most frequent adverse event was nausea, and other complaints included vomiting, headache, dizziness, and leg cramps [43, 46].

SERMs included in the study were raloxifene and bazedoxifene. Both showed a significant effect in preventing secondary fracture. Raloxifene seemed to have a better effect when prescribed at a higher dosage, which was indicated by the significant and substantial heterogeneity between the two groups. Besides beneficial skeletal effects, SERMs reduce the risk of breast cancer [65]. However, an elevated risk of venous thromboembolic events due to raloxifene and bazedoxifene has been described [52]. Additionally, raloxifene significantly raises the risk of discontinuation [50]. Therefore, SERMs should be prescribed with an awareness of their risk of side effects.

Denosumab is a RANKL inhibitor that was proved to possess significant effect on preventing secondary OVCF. Side effects of it include skin rashes, infections, and osteonecrosis of the jaw [62], but presently, there was no significant difference in adverse events compared with control group. Additionally, Boonen et al. reported a significant reduction of fatal adverse events ratio with denosumab in patients with prevalent vertebral fracture [49]. One advantage of denosumab is its low dosing frequency, which might elevate compliance. Romosozumab is a sclerostin inhibitor that has been proved to have better effect on preventing secondary OVCF than alendronate. However, it should be noticed that the cardiac ischemic events and cerebrovascular events ratio were higher in romosozumab group. The role of sclerostin in vessels remains unclear, and the results from basic studies were controversial [66–68]. Therefore, further evaluation of safety profile of romosozumab is needed.

Unlike the superior effects on OVCF of most medications, only zoledronate, risedronate, and PTH had a significant effect on preventing non-vertebral fractures in patients with prevalent OVCF. Combined with the effects of medications on OVCF, the findings might indicate zoledronate, risedronate, and PTH might be better options for patients with prevalent OVCF. Additionally, denosumab and alendronate showed marginally significant effects. The results might have less credibility than the main outcome because of missed information concerning the non-vertebral fracture status of the participants. But, the patients included in this study could still be considered as having a high risk of non-vertebral fracture because prevalent vertebral fracture and low bone mineral density are potential risk factors of non-vertebral fractures [69, 70]. Therefore, the data might be instructive for clinical usage of the medications.

It must be noted that many phase 3 studies were excluded from this meta-analysis because the data of patients with prevalent fractures were not reported. The exclusion might cause an underestimation of the effects of some newly developed medications like denosumab and zoledronate. One limitation of this study include the absence of searching the gray literature, which might increase the risk of publication bias that might lead to an overestimation of the effect of newly developed medications like romosozumab and bazedoxifene. Also, we only included English written manuscript in this study. Though no solid evidence showed a bias caused by the language restriction [71–73], the manuscript written in other languages should be included in further studies for a more comprehensive understanding of the effects of the medications. The generalizability of results of GI complaints was limited, because most of the trials excluded patients with upper GI disease at baseline. Additionally, our criteria for assessing the risk of bias might be too stringent, which might underestimate the quality of evidence. Also, it should be noticed that the most common domains that downregulated the GRADE was the study limitation and imprecision. The same scenario has been reported in a review of systematic reviews, in which the authors indicated the need of high quality RCTs with large sample size for better clinical decisions [74]. In the aspect of study limitation, the two main categories of risk of bias that were rated as unclear to high risk of bias were performance bias and selection bias. For a higher quality of evidence, the report of study might better follow the guidance, like the CONSORT, and report the procedure of randomization and blinding could be great help. To decrease the impact from imprecision, RCTs with higher sample sizes were needed. Also, a report of a subgroup of population with prevalent fracture would help in expanding the sample size.

Most systematic reviews and meta-analyses included osteoporosis patients, regardless their fracture history that introduces indirectness in the results [5–10]. The results might be overestimated on patients had fracture history, and for optimized treatment, accurate analyses of OVCF patients is urged. However, only one systematic review satisfied the demand [11]. Compared with that, we included 14 more RCTs and new medicines such as romosozumab and abaloparatide that allowed for a more comprehensive review and comparisons between different medications. Also, our results included vertebral fracture, non-vertebral fracture, GI complaints of BPs and discontinuation due to AEs. In the end, we evaluated the quality of evidence. The updated information could offer more practical evidence for clinical use.

Our results are consistent with those from other systematic reviews about primary prevention of OVCF [5–7, 9, 75–77]. This could indicate that the medications have a consistent effect on osteoporosis patients, regardless their OVCF history. Also, medications used to prevent osteoporotic fracture had a low risk of severe adverse events in most of the 2–3 years follow-ups. Therefore, the benefits from reducing the risk of fracture, disability, and mortality very likely outweigh the disadvantages. But, careful evaluation of risk factors and arrangement of drug holidays are also necessary to minimize the risk of adverse events [78].

Lack of RCTs that compared interventions of secondary prevention effect limited our assessment of differences between interventions. Although indirect comparisons could be conducted through statistical analyses, high quality RCTs that provide direct evidence are necessary for a solid conclusion.

## Conclusion

High to moderate quality evidence proved zoledronate, alendronate, risedronate, etidronate, ibandronate, PTH, denosumab and SERMs have significant effect on preventing secondary OVCF. Among them, zoledronate, risedronate and PTH also had significant effects on preventing non-vertebra fracture. Moderate quality evidence proved romosozumab had better effect than alendronate. High quality evidence proved that PTH had superior effect to risedronate, but that medication should be prescribed with caution because of its significantly higher risk of adverse events.

## Additional files

Additional file 1: Searching strategy. (DOC 19 kb)

Additional file 2: Risk of bias summary. (DOCX 43 kb)

Additional file 3: Forest plot of secondary outcomes. (DOCX 407 kb)

Additional file 4: Comparison between bisphosphonate. (JPG 1284 kb)

## Data Availability

The datasets used and/or analyzed during the current study are available from the corresponding author on reasonable request.

## Abbreviations

AEs: Adverse events
BPs: Bisphosphonates
BZA: Bazedoxifene
CIs: Confidence intervals
GI: Gastrointestinal
HRT: Hormone replacement therapy
OVCF: Osteoporotic vertebral compression fracture
PTH: Parathyroid hormone
RCTs: Randomized controlled trials
RLX: Raloxifene
SERMs: Selective estrogen receptor modulators
