# Supplementary material for: Effect of medications on prevention of secondary osteoporotic vertebral compression fracture, non-vertebral fracture, and discontinuation due to adverse events: a meta-analysis of randomized controlled trials
Source: BMC Musculoskelet Disord. 2019 Aug 31;20:399. doi: 10.1186/s12891-019-2769-8 (PMC6717630; doi:10.1186/s12891-019-2769-8)
Supplement: Supplementary file 1 — Searching strategy. (DOC 19 kb) [file 12891_2019_2769_MOESM1_ESM.doc]

PubMed:

1. randomized controlled trial[pt]
2. controlled clinical trial[pt]
3. randomized controlled trials[mh]
4. random allocation[mh]
5. double-blind method[mh]
6. single blind method[mh]
7. clinical trial[pt]
8. clinical trials[mh])
9. clinical trial"[tw]
10. latin square[tw])
11. placebos[mh]
12. placebo*[tw]
13. random*[tw]
14. research design[mh:noexp]
15. placebos[mh]
16. control*[tw]
17. prospective*[tw]
18. volunteer*[tw]) NOT (animal[mh] NOT human[mh])
19. #1 OR #2 OR #3 OR #4 OR #5 OR #6 OR #7 OR #8 OR #9 OR #10 OR #11 OR #12 OR #13 OR #14 OR #15 OR #16 OR #17 OR #18 OR #19
20. Osteoporosis compression fracture
21. osteoporotic fracture
22. #20 OR #21
23. spine
24. spinal
25. vertebral
26. vertebrae
27. #23 OR #24 OR #25 OR #26
28. medication[tiab]
29. medicine[tiab]
30. drug*[tiab]
31. bisphosphonate[tiab]
32. bisphosphonates[tiab]
33. alendronate[tiab]
34. risedronate[tiab]
35. ibandronate[tiab]
36. clodronate[tiab]
37. zoledronate[tiab]
38. pamidronate[tiab]
39. parathyroid hormone[tiab]
40. parathyroid hormone[tw]
41. teriparatide[tiab]
42. teriparatide[tw]
43. abaloparatide[tiab]
44. alaloparatide[tw]
45. denosumab[tiab])
46. denosumab[tw]
47. calcium[tiab]
48. vitamine D[tiab]
49. calcitonin[tiab]
50. calcitonin[tw]
51. serm[tiab]
52. raloxifene[tiab])
53. bazedoxifene[tiab]
54. HRT[tiab]
55. HRT[tw]
56. ERT[tiab]
57. ERT[tw]
58. hormone replacement[tiab]
59. hormone replacement[tw]
60. estrogen replacement[tiab]
61. estrogen replacement[tw]
62. estradiol[tiab]
63. estradiol[tw]
64. estrone[tiab]
65. estrone[tw]
66. dien estrol[tiab]
67. dien estrol[tw]
68. pamidronate[tiab]
69. romosozumab[tiab]
70. romosozumab[tw]
71. #28 OR #29 OR #30 OR #31 OR #32 OR #33 OR #34 OR #35 OR #36 OR #37 OR #38 OR #39 OR #40 OR #41 OR #42 OR #43 OR #44 OR #45 OR #46 OR #47 OR #48 OR #49 OR #50 OR #51 OR #52 OR #53 OR #54 OR #55 OR #56 OR #57 OR #58 OR #59 OR #60 OR #61 OR #62 OR #63 OR #64 OR #65 OR #66 OR #67 OR #68
72. #19 AND #22 AND #27 AND #71

Cochrane library

#1 MeSH descriptor: [Osteoporosis] explode all trees

#2 MeSH descriptor: [Spinal Fractures] explode all trees

#3 MeSH descriptor: [Spinal Cord Compression] explode all trees

#4 MeSH descriptor: [Spinal Fractures] explode all trees

#5 vertebral near (compression or fracture)

#6 (thoracic or lumbar or spin*) near fracture*

#7 compression near fracture

#8 (thoracic or lumbar or spin*) near compression

#9 "fractured vertebrae"

#10 bisphosphonate or bisphosphonates or alendronate or risedronate or ibandronate or clodronate or zoledronate or pamidronate or parathyroid hormone or abaloparatide or denosumab or calcium or vitamine D or calcitonin or serm or raloxifene or bazedoxifene or hormone replacement or hrt or ert or estrogen or estrodiol or estrol or romosozumab

#11 #2 or #3 or #4 or #5 or #6 or #7 or #8 or #9

#12 #11 and #10
