# Supplementary material for: Effect of medications on prevention of secondary osteoporotic vertebral compression fracture, non-vertebral fracture, and discontinuation due to adverse events: a meta-analysis of randomized controlled trials
Source: BMC Musculoskelet Disord. 2019 Aug 31;20:399. doi: 10.1186/s12891-019-2769-8 (PMC6717630; doi:10.1186/s12891-019-2769-8)
Supplement: Supplementary file 2 — Risk of bias summary. (DOCX 43 kb) [file 12891_2019_2769_MOESM2_ESM.docx]

Additional file 2. Risk of bias table

| Article ID | Year | a | b | c | d | e | f | g | h | i | j | k | l | m |
| --- | --- | --- | --- | --- | --- | --- | --- | --- | --- | --- | --- | --- | --- | --- |
| **Zoledronic acid** |  |  |  |  |  |  |  |  |  |  |  |  |  |  |
| Nakamura | 2017 | + | + | + | ? | ? | + | ? | + | + | + | + | + | + |
| **Alendronate** |  |  |  |  |  |  |  |  |  |  |  |  |  |  |
| Black | 1996 | + | + | + | + | + | + | + | + | + | + | + | + | + |
| Kushida | 2004 a | ? | ? | + | ? | ? | + | ? | + | + | + | + | + | ? |
| Liberman | 1995 | ? | ? | + | - | - | + | ? | + | + | + | + | + | ? |
| **Risedronate** |  |  |  |  |  |  |  |  |  |  |  |  |  |  |
| Clemmesen | 1997 | ? | ? | + | ? | ? | + | + | + | + | ? | + | + | - |
| Fogelman | 2000 | ? | ? | + | ? | ? | + | ? | + | + | + | + | + | + |
| Harris | 1999 | + | + | + | + | + | + | + | + | + | ? | + | + | + |
| Reginster | 2000 | ? | ? | + | ? | ? | + | + | + | + | + | ? | + | ? |
| Sorensen | 2003 | ? | ? | - | ? | ? | ? | ? | + | + | + | + | + | ? |
| **Etidronate** |  |  |  |  |  |  |  |  |  |  |  |  |  |  |
| Guanabens | 2000 | ? | ? | + | ? | ? | ? | - | + | + | ? | ? | + | ? |
| Harris | 1993 | + | ? | + | ? | ? | + | ? | + | + | + | + | + | ? |
| Lyritis | 1997 | ? | ? | + | - | - | + | ? | + | + | + | ? | + | ? |
| Montenssori | 1997 | + | ? | + | ? | ? | ? | ? | + | + | + | + | + | ? |
| Shiota | 2001 | ? | ? | + | ? | ? | ? | ? | + | + | + | ? | ? | ? |
| Watts | 2014 | + | ? | + | ? | ? | + | ? | + | + | + | ? | + | ? |
| Wimalawansa | 1998 | + | ? | + | ? | ? | + | ? | + | + | + | ? | + | ? |
| **Ibandronate** |  |  |  |  |  |  |  |  |  |  |  |  |  |  |
| Chesnut | 2004 | + | ? | + | ? | ? | + | ? | + | + | ? | + | + | + |
| Recker | 2004 | + | + | + | ? | ? | + | + | + | + | + | + | + | ? |
| **Minodronate** |  |  |  |  |  |  |  |  |  |  |  |  |  |  |
| Matsumoto | 2009 | + | + | + | ? | ? | + | + | + | + | ? | + | + | ? |
| **Pamidronate** |  |  |  |  |  |  |  |  |  |  |  |  |  |  |
| Brumsen | 2002 | ? | ? | + | + | + | + | + | + | + | + | + | + | + |
| **Calcitonin** |  |  |  |  |  |  |  |  |  |  |  |  |  |  |
| Chesnut | 2005 | + | ? | + | ? | ? | ? | + | + | + | + | + | + | + |
| Hodsman | 1997 | ? | ? | + | ? | ? | + | ? | + | + | + | ? | + | ? |
| Peichl | 1999 | ? | ? | ? | - | - | ? | ? | + | + | ? | ? | + | ? |
| **HRT** |  |  |  |  |  |  |  |  |  |  |  |  |  |  |
| Gutteridge | 2002 | + | ? | ? | - | - | + | ? | + | + | + | ? | + | ? |
| Lufkin | 1992 | ? | ? | + | + | + | + | ? | + | + | + | ? | ? | ? |
| **PTH** |  |  |  |  |  |  |  |  |  |  |  |  |  |  |
| Fujita | 2014 | + | + | + | ? | ? | + | ? | + | + | + | + | + | + |
| Greenspan | 2007 | + | + | + | ? | ? | + | - | + | + | ? | + | + | + |
| Nakamura | 2012 | + | + | + | + | + | + | ? | + | + | + | + | + | + |
| Neer | 2001 | ? | ? | + | ? | ? | + | + | + | + | + | ? | + | + |
| Cosman | 2018 | + | + | + | + | + | + | + | + | + | + | + | + | + |
| **Denosumab** |  |  |  |  |  |  |  |  |  |  |  |  |  |  |
| Boonen | 2011 | ? | ? | + | ? | ? | + | ? | + | + | + | + | + | + |
| Nakamura | 2014 | ? | ? | + | ? | ? | + | ? | + | + | + | + | + | ? |
| **SERMs** |  |  |  |  |  |  |  |  |  |  |  |  |  |  |
| Palacios | 2015 | + | + | + | ? | ? | - | + | + | + | ? | + | + | + |
| Ettinger | 1999 | + | + | + | + | + | + | + | + | + | + | + | + | + |
| Lufkin | 1998 | + | ? | + | ? | ? | + | ? | + | + | + | + | + | ? |
| **Between medication** | |  |  |  |  |  |  |  |  |  |  |  |  |  |
| **Ibandronate vs. Risedronate** | | | |  |  |  |  |  |  |  |  |  |  |  |
| Ito | 2017 | + | + | + | + | + | + | + | + | + | + | + | + | + |
| **Risedronate vs. Etidronate** | | | |  |  |  |  |  |  |  |  |  |  |  |
| Kushida | 2004 b | ? | ? | + | + | + | + | ? | + | + | + | ? | + | ? |
| **Risedronate vs. Teriparatide** | | | |  |  |  |  |  |  |  |  |  |  |  |
| Hadji | 2012 | ? | ? | ? | + | ? | + | ? | + | + | + | + | + | ? |
| Kendler | 2017 | + | + | + | + | + | + | + | + | + | + | + | + | + |
| **Monoclonal antibody vs. Alendronate** | | | | | |  |  |  |  |  |  |  |  |  |
| Nakamura | 2014 | ? | ? | + | ? | ? | + | ? | + | + | + | + | + | ? |
| Saag | 2017 | + | + | + | ? | ? | + | ? | + | + | + | + | + | + |
| **Etidronate vs. HRT** | |  |  |  |  |  |  |  |  |  |  |  |  |  |
| Wimalawansa | 1998 | + | ? | + | ? | ? | + | ? | + | + | + | ? | + | ? |

a, random sequence generation (selection bias)

b, allocation concealment (selection bias)

c, group similarity at baseline (selection bias)

d, blinding to patients (performance bias)

e, blinding to care providers (performance bias)

f. influence of co-interventions (performance bias)

g, compliance with interventions (performance bias)

h, blinding to outcome assessors (detection bias) - Fracture

i, timing of outcome assessments (detection bias)

j, incompleter outcome data (attribution bias) - lost ratio

k, incomplete outcome data (attribution bias) - ITT or modified ITT

l, selective reporting (reporting bias)

m, other source of bias

Reasons of being rated as unclear or high risk of bias.

| Article ID | Year | Reasons |
| --- | --- | --- |
| **Zoledronic acid** |  |  |
| Nakamura | 2017 | d, Not reported; e, Not reported; g, Not reported; |
| **Alendronate** |  |  |
| Black | 1996 |  |
| Kushida | 2004 a | a, Not reported; b, Not reported; d, Not reported; e, Not reported; g, Not reported; m, Conflict of interest was not stated. |
| Liberman | 1995 | a, Not reorted; b, Not reported; d, Open label; e, Open label; g, Not reported; m, Conflict of interest was not stated. |
| **Risedronate** |  |  |
| Clemmesen | 1997 | a, Not reported; b, Not reported; d, No description of the appreance of geltain capsules; e, Not reported; j: 32% i n3 years; m, the criteria of fracture were different in different centers. |
| Fogelman | 2000 | a, Not reported; b, Not reported; d, Not reported; e, Not reported; g, Not reported; j, 21% in 2 years; n, no conlifct of interest stated. |
| Harris | 1999 | j, 42% in 3 years. |
| Reginster | 2000 | a, Not reported; b, Not reported; d, Not reported; e, Not reported;, k, Not reported; m, Not reported. |
| Sorensen | 2003 | a, Not reported; b, Not reported; c, Participants in risedronate group already had 3 years risedronate; d, Not reported; e, Not reported; g, Not eported; m, Conflict of interest was not stated. |
| **Etidronate** |  |  |
| Guanabens | 2000 | a, Not reported; b, Not reported; d: Not reported; e, Not reported; f, Participants in etidronate group did not receive calcium on the day they receive etidronate; g, Significant (p=0.01) difference in complaince of medications between groups; j, 34% in 3 years; k, no report of adverse events; m, no conflict of interest reported. |
| Harris | 1993 | b, Not reported; d, Not reported; e, Not reported; g, Not reported; m, Conflict of interest was not stated. |
| Lyritis | 1997 | a, Not reported; b, Not reported; d, Open label design; e, Open label design; g, Not reported; k, Not reported; m, Conflict of interest is not stated. |
| Montenssori | 1997 | b, Not reported; d, Not reported; e, Not reported; f, Calcium intake is different between groups; g, Not reported; m, Not reported |
| Shiota | 2001 | a, Not reported; b, Not reported; d, Not reported; e, Not reported; f, Calcium and alfacalcidol supplement were different between groups; g, Not reported; k, Not reported;l, Safety data was not reported; m, Conflict of interest was not stated. |
| Watts | 2014 | b, Not reported; d, Not reported; e, Not reported; g, Not reported; k, Not reported; m Conflict of interest was not stated. |
| Wimalawansa | 1998 | b, Not reported; d, Not reported; e, Not reported; g, Not reported; k, Not reported; m, Conflict of interest was not stated. |
| **Ibandronate** |  |  |
| Chesnut | 2004 | b, Not reported; d, Not reported; e, Not reported; j, 34% in 3 years. |
| Recker | 2004 | d, Not reported; e, Not reported; g, Not reported; e, Not reported; j, Not reported. |
| **Minodronate** |  |  |
| Matsumoto | 2009 | d, Not reported; e, Not reported; j, 30.11% in 3 years. |
| **Pamidronate** |  |  |
| Brumsen | 2002 | a, Not reported; b, Not reported. |
| **Calcitonin** |  |  |
| Chesnut | 2005 | b, Not reported; d, Not reported; e, Not reported. |
| Hodsman | 1997 | a, Not reported; b, Not reported; d, Not reported; e, Not reported; g, Not reported; j, 23% in 2 years; k, Not reported; m,, Conflict of interest was not stated. |
| Peichl | 1999 | a, Not reported; b, Not reported; c, Concommitant medication history is different between participants; d, Open design; e, Open design; f, Vitamin D is only offered for control group;  g, Not reported; j, Not reported; k, Not reported; m, Conflict of interest is not stated. |
| **HRT** |  |  |
| Gutteridge | 2002 | b, Not reported; b, Not reported; c, Age and CaE at the baseline is different between groups; d, Open design; e, Open design;  g, Not reported; j, 24% in 2 years; k, Not reported; m, Conflict of interest is not stated. |
| Lufkin | 1992 | b, Not reported; b, Not reported; g, Not reported; j, Not reported; m, Conflict of interest was not stated. |
| **PTH** |  |  |
| Fujita | 2014 | d, No specific description; e, No specific description; g, Not reported. |
| Greenspan | 2007 | c, Not reported; d, Not reported; g; Compliance was different among different groups.j, 32.82% in 18 months. |
| Nakamura | 2012 | g, Not reported. |
| Neer | 2001 | a, Not reported; b, Not reported; d, Not reported; e, Not reporeted; j, Not reported. |
| **Denosumab** |  |  |
| Boonen | 2011 | a, Not reported; b, Not reported; d, Not reported; e, Not reported; g, 5979 (76% received all injections (Cummings, 2009). |
| Nakamura | 2014 | a, Not reported; b, Not reported; d, Not reported; e, Not reported; g, Not reported; m, Sponsors were resposible for data collection and analysis.. |
| **SERMs** |  |  |
| Palacios | 2015 | d, Not reported; e, Not reported; f, Significantly (p<0.01) higher proportion of Placebo-treated women used concomitant bone-active nonstudy mdications; j, 25% in extension 2; 77% in whole trial ( 7 years). |
| Ettinger | 1999 |  |
| Lufkin | 1998 | b, Not reported; d, Not reported; e, Not reported; g, Not reported; j, Not reported; m, Conflict of interest is not stated. |
| **Between medication** | |  |
| **Ibandronate vs. Risedronate** | |  |
| Nakamura | 2013 |  |
| **Risedronate vs. Etidronate** | |  |
| Kushida | 2004 b | a, Not reported; b, Not reported; g, Not reported; k, 23% in 2 years; k, per protocol set was used; m, Conflict of interest was not stated. |
| **Risedronate vs. Teriparatide** | |  |
| Hadji | 2012 | a, Not reported; b, Not reported; c, BMD of femoral neck is different between groups; f, Not reported; g, Not reported; j, 26% in 18 months; n, Data was collected and analyzed by sponsor. |
| Kendler | 2017 |  |
| **Monoclonal antibody vs. Alendronate** | | |
| Nakamura | 2014 | a, Not reported; b, Not reported; d, Not reported; e, Not reported; g, Not reported; m, Sponsors were resposible for data collection and analysis.. |
| Saag | 2017 | d, Not reported; e, Not reported; g, Not reported; h, Not reported. |
| **Etidronate vs. HRT** | |  |
| Wimalawansa | 1998 | b, Not reported; d, Not reported; e, Not reported; g, Not reported; k, Not reported; m, Conflict of interest was not stated. |
