# Supplementary material for: Effect of medications on prevention of secondary osteoporotic vertebral compression fracture, non-vertebral fracture, and discontinuation due to adverse events: a meta-analysis of randomized controlled trials
Source: BMC Musculoskelet Disord. 2019 Aug 31;20:399. doi: 10.1186/s12891-019-2769-8 (PMC6717630; doi:10.1186/s12891-019-2769-8)

[a. Forest plot. Effect of antiresorptive medications preventing secondary vertebral fracture. 3](#_Toc501984025)

[b. Forest plot. GI complaints of bisphosphonates. 4](#_Toc501984026)

[c. Forest plot. Discontinuation due to AEs – Zoledronate 5](#_Toc501984027)

[d. Forest plot. Preventing non-vertebral fracture – Zoledronate 5](#_Toc501984028)

[e. Forest plot. GI complaints - Alendronate 5](#_Toc501984029)

[f. Forest plot. Discontinuation due to AEs – Alendronate 5](#_Toc501984030)

[g. Forest plot. Non-vertebral fracture – Alendronate 6](#_Toc501984031)

[h. Forest plot. GI complaints – Risedronate 7](#_Toc501984032)

[i. Forest plot. Discontinuation due to AEs - Risedronate 7](#_Toc501984033)

[j. Forest plot. Non-vertebral fracture – Risedronate 7](#_Toc501984034)

[k. Forest plot. Sensitivity test. Excluding a study with a small sample size and big variance with other studies. 8](#_Toc501984035)

[l. Forest plot. GI complaints – Etidronate 8](#_Toc501984036)

[m. Forest plot. Discontinuation – Etidronate 8](#_Toc501984037)

[n. Forest plot. Non-vertebral fracture – Etidronate 9](#_Toc501984038)

[o. Forest plot. Discontinuation due to AEs – Ibandronate (sufficient dose) 9](#_Toc501984039)

[p. Forest plot. Discontinuation due to AEs – Ibandronate (insufficient dose) 9](#_Toc501984040)

[q. Forest plot. Non-vertebral fracture – Ibandronate (sufficient dose) 9](#_Toc501984041)

[r. Forest plot. Non-vertebral fracture – Ibandronate (insufficient dose) 10](#_Toc501984042)

[s. Forest plot. Non-vertebral fracture – Minodronate 10](#_Toc501984043)

[t. Forest plot. Non-vertebral fracture – Pamidronate 10](#_Toc501984044)

[u. Forest plot. Non-vertebral fracture – HRT 10](#_Toc501984045)

[v. Forest plot. Discontinuation due to AEs – HRT 11](#_Toc501984046)

[w. Forest plot. Discontinuation due to AEs – PTH 11](#_Toc501984047)

[x. Forest plot. Non-vertebral fracture – PTH 12](#_Toc501984048)

[y. Forest plot. Discontinuation due to AEs – Denosumab 12](#_Toc501984049)

[z. Forest plot. Non-vertebral fracture – Denosumab 12](#_Toc501984050)

[aa. Forest plot. Discontinuation – Risedronate vs. PTH 13](#_Toc501984051)

[bb. Forest plot. Non-vertebral fracture – Teriparatide vs. Risedronate 13](#_Toc501984052)

[cc. Forest plot. Non-vertebral fracture – Alendronate vs. Denosumab 13](#_Toc501984053)

[cc. Forest plot. Non-vertebral fracture – Romosozumab vs. Alendronate 13](#_Toc501984054)

# a. Forest plot. Effect of antiresorptive medications preventing secondary vertebral fracture.


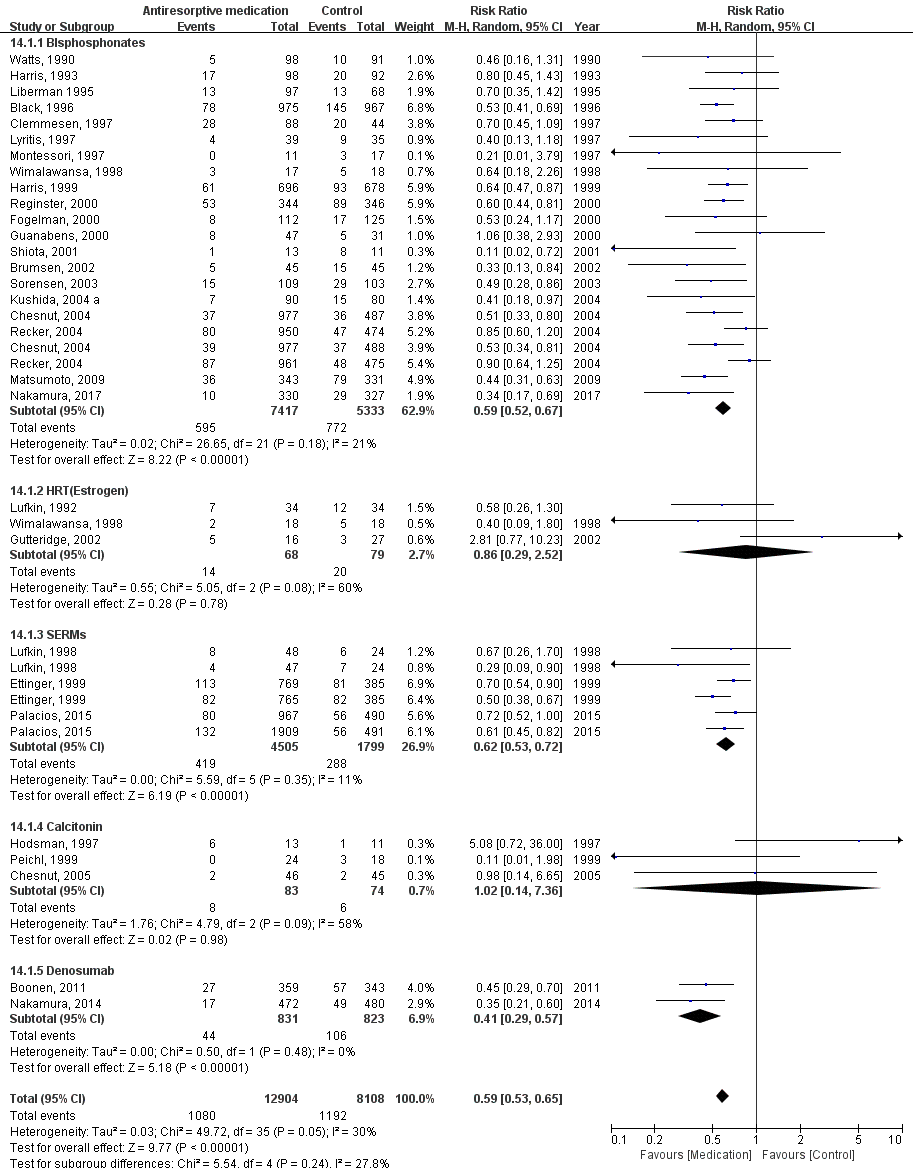


# b. Forest plot. GI complaints of bisphosphonates.


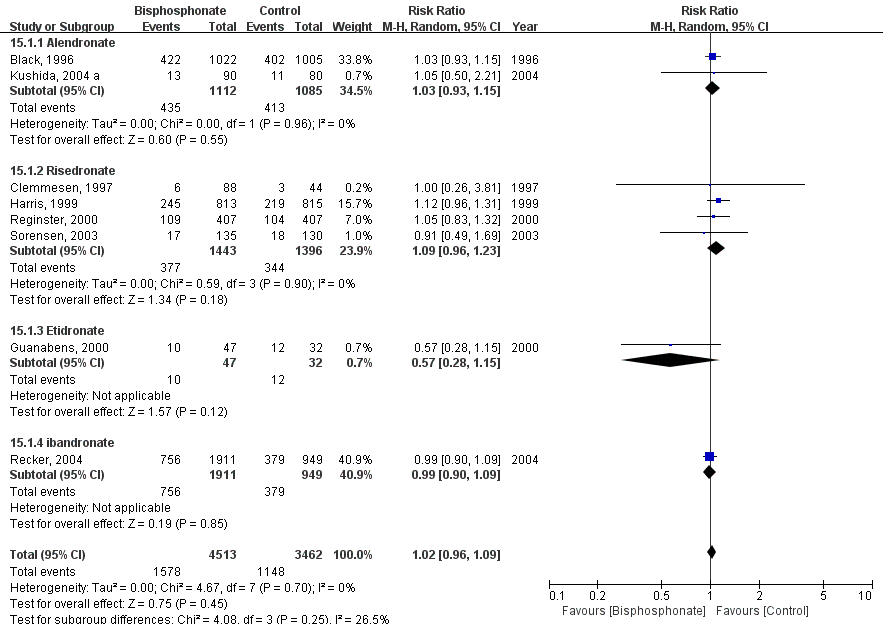


# c. Forest plot. Discontinuation due to AEs – Zoledronate


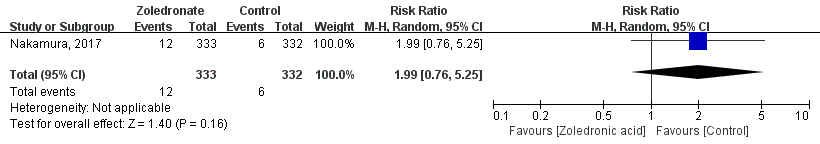


# d. Forest plot. Preventing non-vertebral fracture – Zoledronate


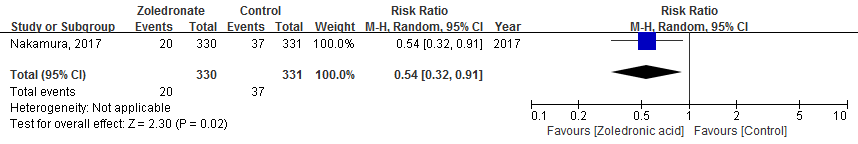


# e. Forest plot. GI complaints - Alendronate


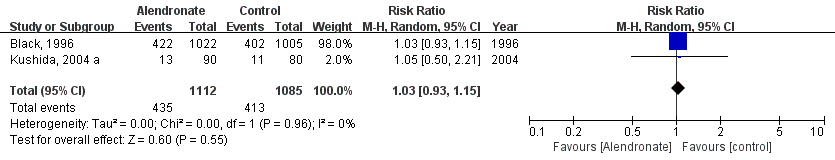


# f. Forest plot. Discontinuation due to AEs – Alendronate


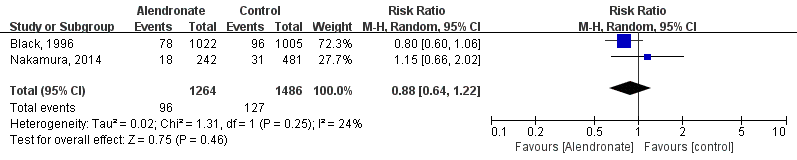


# g. Forest plot. Non-vertebral fracture – Alendronate


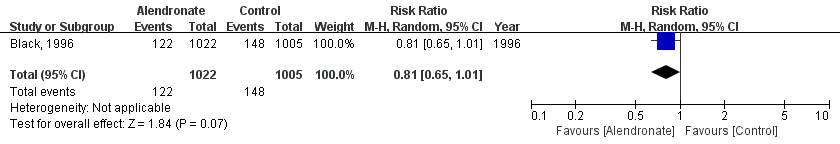


# h. Forest plot. GI complaints – Risedronate


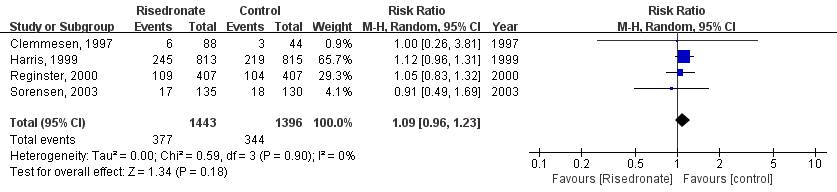


# i. Forest plot. Discontinuation due to AEs - Risedronate


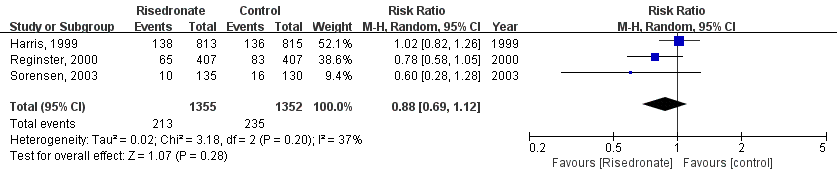


# j. Forest plot. Non-vertebral fracture – Risedronate


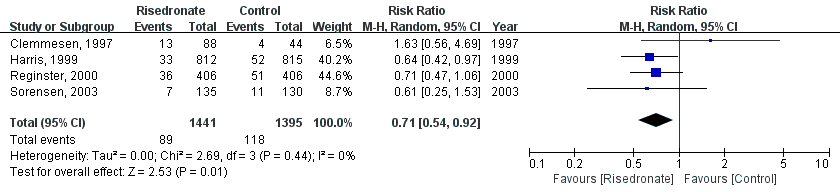


# k. Forest plot. Sensitivity test. Excluding a study with a small sample size and big variance with other studies.


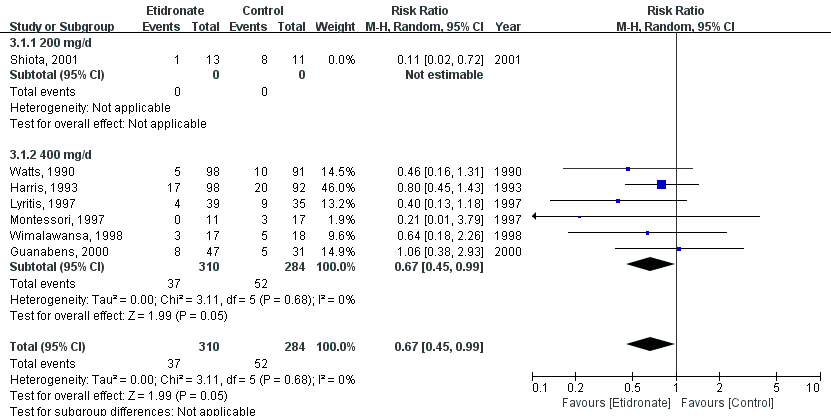


# l. Forest plot. GI complaints – Etidronate


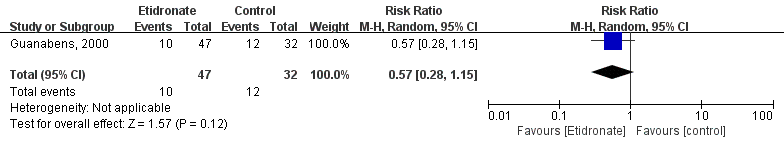


# m. Forest plot. Discontinuation – Etidronate


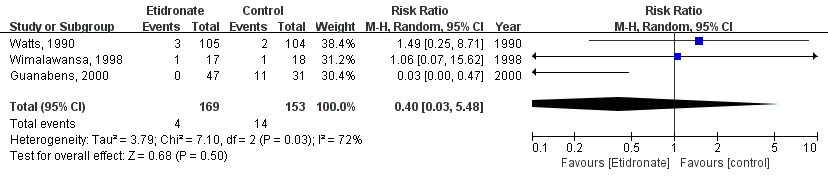


# n. Forest plot. Non-vertebral fracture – Etidronate


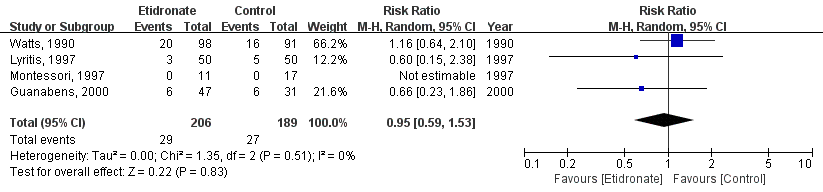


# o. Forest plot. Discontinuation due to AEs – Ibandronate (sufficient dose)


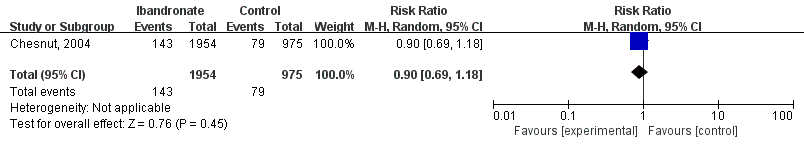


# p. Forest plot. Discontinuation due to AEs – Ibandronate (insufficient dose)


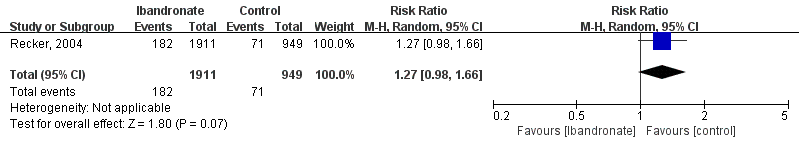


# q. Forest plot. Non-vertebral fracture – Ibandronate (sufficient dose)


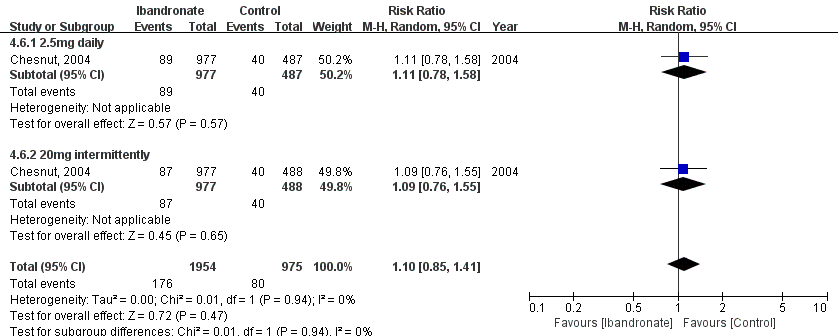


# r. Forest plot. Non-vertebral fracture – Ibandronate (insufficient dose)


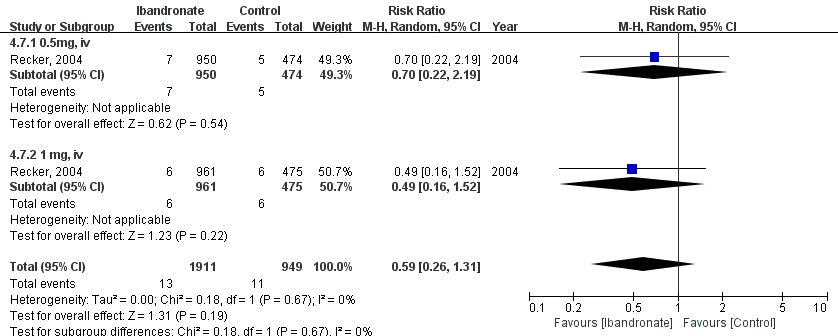


# s. Forest plot. Non-vertebral fracture – Minodronate


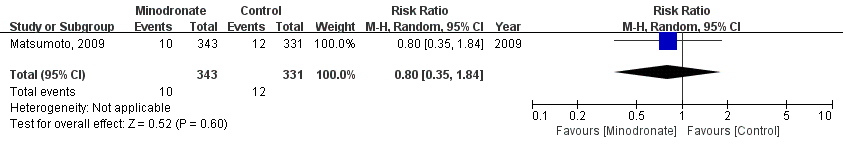


# t. Forest plot. Non-vertebral fracture – Pamidronate


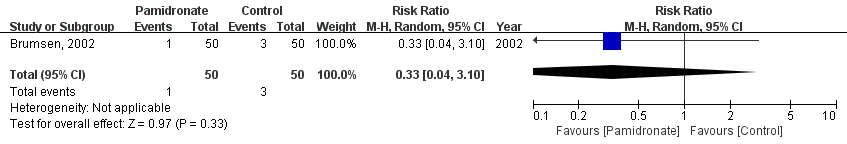


# u. Forest plot. Non-vertebral fracture – HRT


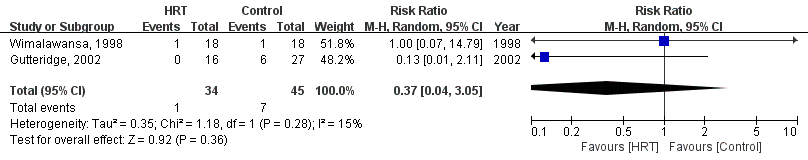


# v. Forest plot. Discontinuation due to AEs – HRT


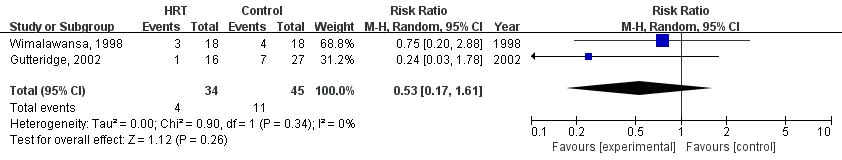


# w. Forest plot. Discontinuation due to AEs – PTH


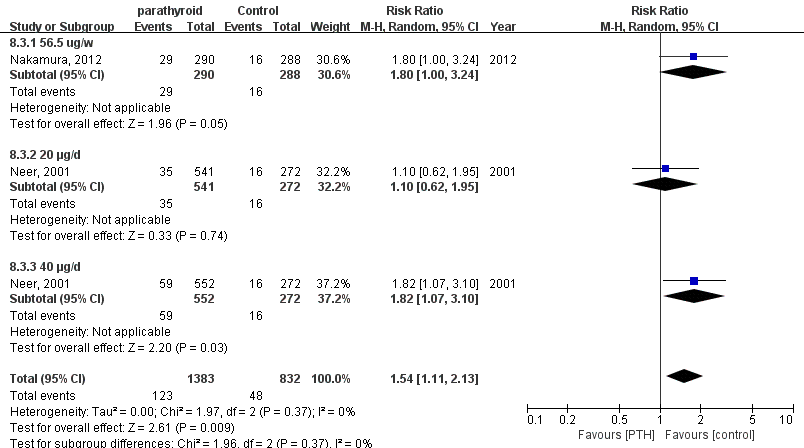


# x. Forest plot. Non-vertebral fracture – PTH


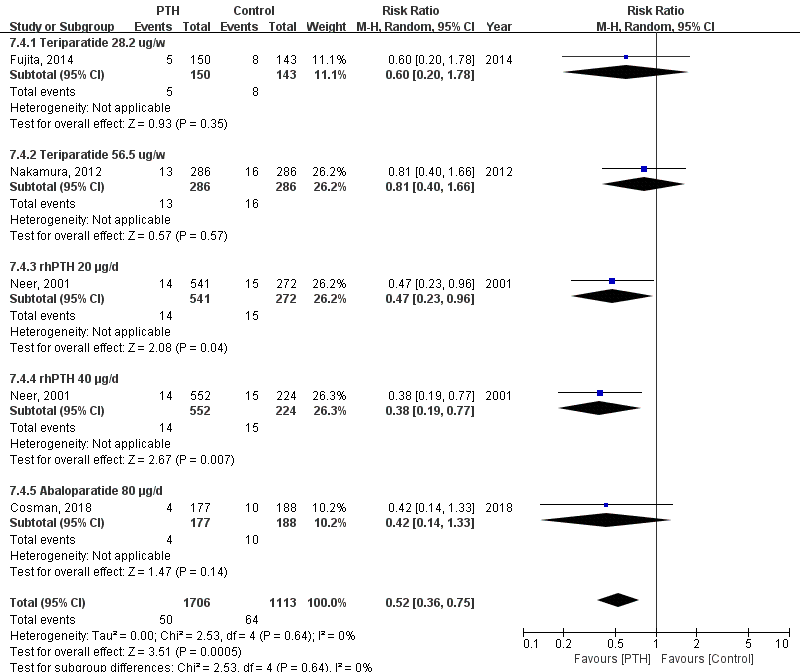


# y. Forest plot. Discontinuation due to AEs – Denosumab


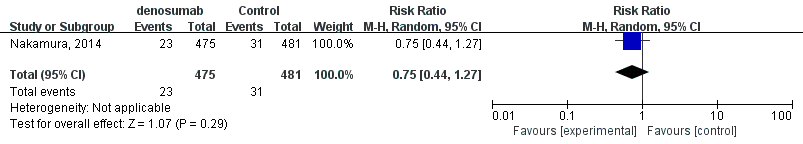


# z. Forest plot. Non-vertebral fracture – Denosumab


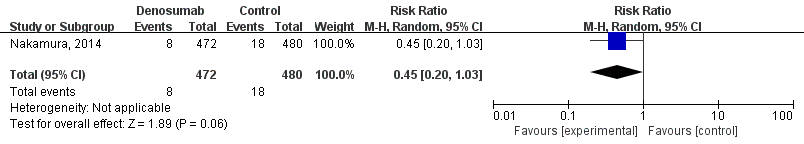


# aa. Forest plot. Non-vertebral fracture – Ibandronate vs. Risedronate


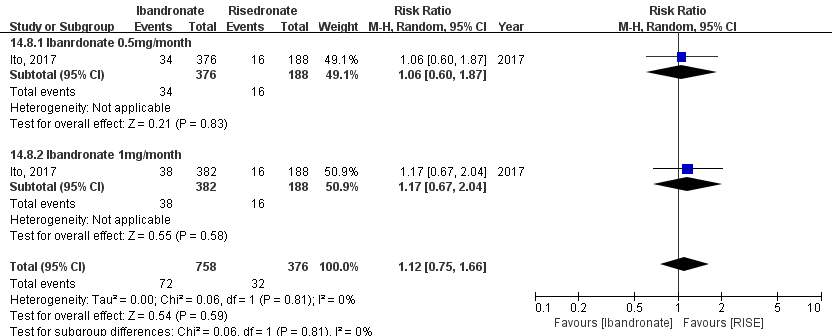


# bb. Forest plot. Discontinuation – Risedronate vs. PTH


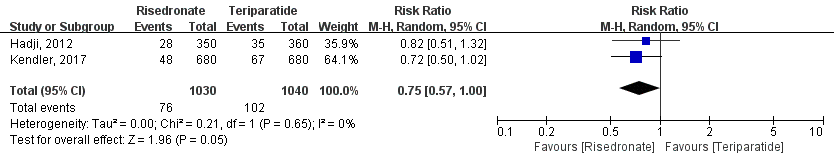


# cc. Forest plot. Non-vertebral fracture – Teriparatide vs. Risedronate


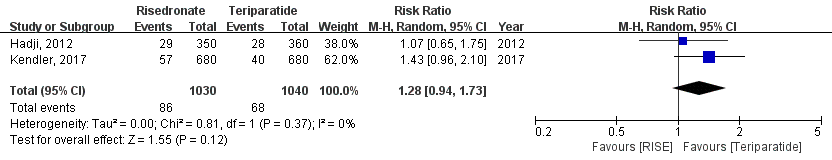


# dd. Forest plot. Non-vertebral fracture – Alendronate vs. Denosumab


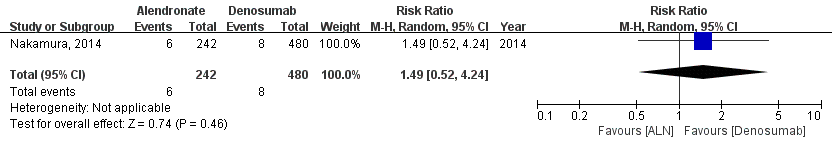


# ee. Forest plot. Non-vertebral fracture – Romosozumab vs. Alendronate


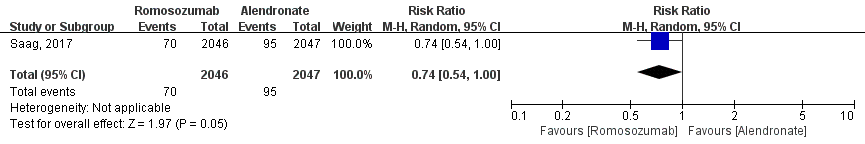

Supplement: Supplementary file 3 — Forest plot of secondary outcomes. (DOCX 407 kb) [file 12891_2019_2769_MOESM3_ESM.docx]
