# Supplementary figures and images for: Effect of medications on prevention of secondary osteoporotic vertebral compression fracture, non-vertebral fracture, and discontinuation due to adverse events: a meta-analysis of randomized controlled trials
Source: BMC Musculoskelet Disord. 2019 Aug 31;20:399. doi: 10.1186/s12891-019-2769-8 (PMC6717630; doi:10.1186/s12891-019-2769-8)

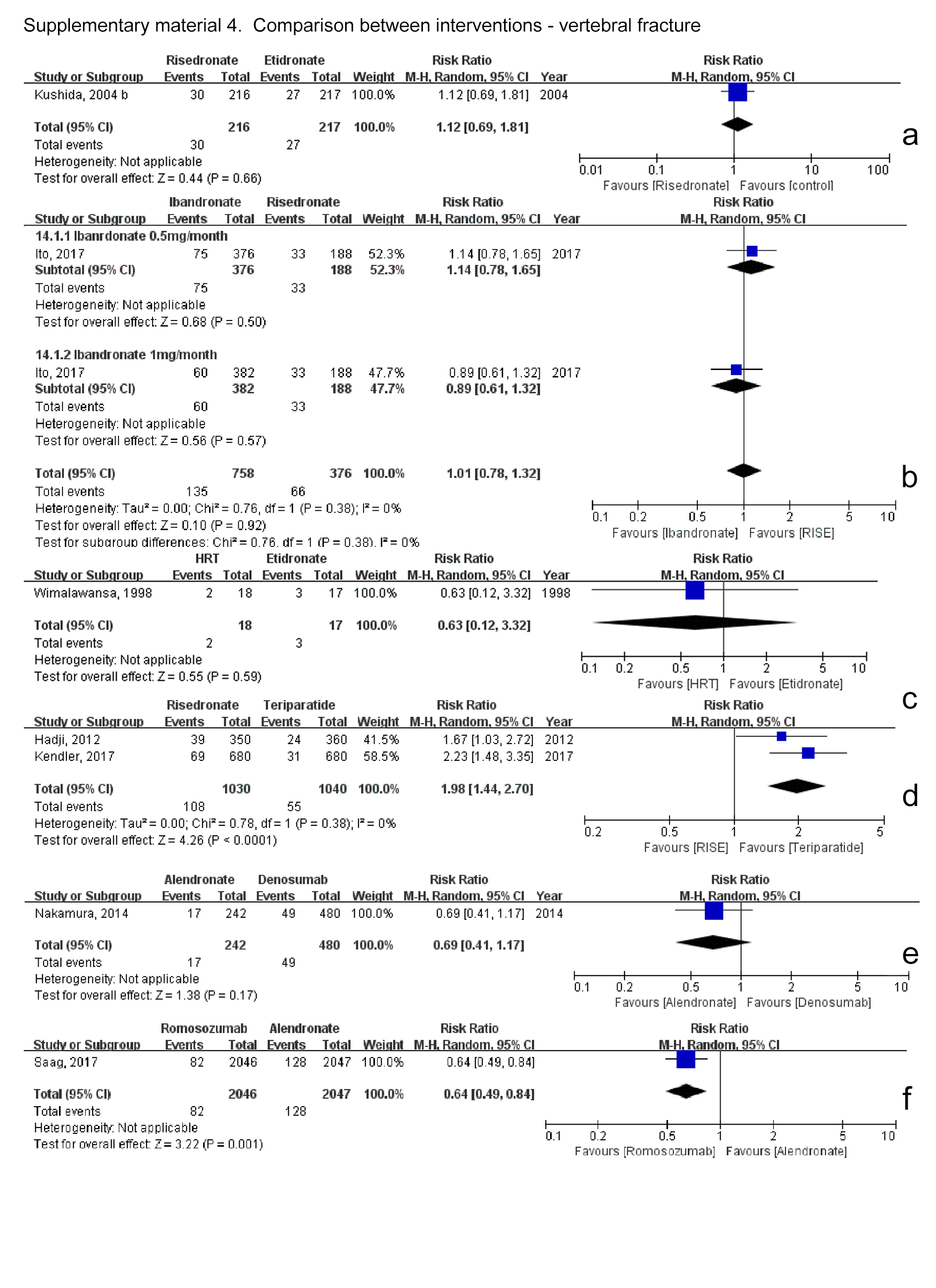

Supplement: Supplementary file 4 — Comparison between bisphosphonate. (JPG 1284 kb) [file 12891_2019_2769_MOESM4_ESM.jpg]
